# Multi-proteomics reveals integrated metabolic and regulatory networks for xylan catabolism in *Streptomyces* sp. SirexAA-E

**DOI:** 10.1128/spectrum.02251-25

**Published:** 2025-11-20

**Authors:** Tatsuya Nagano, Keisuke Ohashi, Petra Banko, Vijay Kumar, Chiaki Hori, Brian G. Fox, Taichi E. Takasuka

**Affiliations:** 1 Graduate School of Global Food Resources, Hokkaido University 12810, Sapporo, Japan; 2 Research Faculty of Agriculture, Hokkaido University 12810, Sapporo, Japan; 3 Department of Microbiology, Guru Nanak Dev University 29740, Amritsar, Punjab, India; 4 Environmental Molecular Biology Section of Environmental Biology, Faculty of Environmental Earth Science, Hokkaido University 12810, Sapporo, Japan; 5 Department of Biochemistry, University of Wisconsin-Madison 5228, Madison, Wisconsin, USA; 6 Global Station for Food, Land and Water Resources, Hokkaido University 12810, Sapporo, Japan; Universitat Wien, Vienna, Austria

**Keywords:** *Streptomyces *sp. SirexAA-E, xylan utilization, sugar transporter, TMT proteomics, transcriptional regulator

## Abstract

An insect-associated bacterium, *Streptomyces* sp. SirexAA-E (SirexAA-E) secretes plant biomass-degrading enzymes depending on the available cell wall materials. SirexAA-E readily utilizes xylan, one of the major hemicelluloses. However, the enzyme composition and pathways supporting xylan metabolism in SirexAA-E and other Streptomycetes have not been reported. We aimed to understand changes in both extracellular and intracellular protein productions by cultivating SirexAA-E in defined media containing either xylose, xylobiose, or xylan. Proteomics showed each carbon source gave specific extracellular protein composition when compared to glucose. Furthermore, intracellular proteomics using a pair-wise Tandem Mass Tag (TMT)-labeling LC-MS/MS identified 1,037 proteins with changes in the xylose-, xylobiose-, or xylan-dependent proteome relative to the glucose-derived proteome. We found that numerous proteins related to oxidative phosphorylation were enriched in the pentose-grown proteomes relative to the glucose proteome. This observation is consistent with a high demand for ATP to support protein secretion and intake of xylose and xylooligosaccharides. Additionally, several transporters for carbon uptake were identified in the genome of SirexAA-E by protein homology comparison with *Streptomyces coelicolor*, and several key transcriptional regulators used by SirexAA-E for catabolizing xylan were found by pull-down proteomics. The current study provides new insights into the extensive extracellular and intracellular responses of a cellulolytic *Streptomyces* to the major plant hemicellulose.

**IMPORTANCE:**

*Streptomyces* sp. SirexAA-E can efficiently degrade cellulose, xylan, and mannan, the major polysaccharide components of woody biomass. Our previous work showed the relative simplicity of the secreted proteome used to degrade cellulose. In this study, we report on the extracellular and intracellular proteomic responses of SirexAA-E during growth on xylan. The substrate-specific proteomic profiles have given a new understanding of the regulation of xylanolytic enzymes and additional metabolic pathways supporting growth on a pentose sugar. These groupings of regulatory and structural proteins provide a blueprint for construction of more robust strains for biomass valorization.

## INTRODUCTION

*Streptomyces* sp. SirexAA-E (SirexAA-E) was originally isolated as one of the symbionts of the pine-boring woodwasp, *Sirex noctilio*, and it is known that SirexAA-E and other cellulolytic bacteria help the larvae of *S. noctilio* utilize polysaccharides (1). SirexAA-E decomposes both cellulosic and hemicellulosic materials and shows high plant biomass-degrading potential as we have shown it secretes various combinations of cellulose and hemicellulose-degrading enzymes in response to available carbon sources (2). Hence, understanding the mechanism of polysaccharide-specific protein expression in this bacterium may enable genome engineering of related industrial strains for improved plant biomass decomposition (3). To this end, several transcriptional regulators of cellulases or hemicellulases have been characterized in SirexAA-E. For example, a major cellulose-responsive regulator, SsCebR, was identified, which regulates at least 15 different genes encoding for cellulose-utilizing carbohydrate-active enzymes (CAZymes) (https://www.cazy.org/) (2, 4), including highly secreted β−1,4-cellobiohydrolases SACTE_0236 (GH48) and SACTE_0237 (GH6), β−1,4-mannanase, SACTE_2347 (GH5) (5), lytic polysaccharide monooxygenase (LPMO), SACTE_3159 (AA10) (6), and carbohydrate esterases (CEs) (5, 7). The pattern of secreted enzymes in the culture containing cellulose was similar in cellobiose-grown culture but was distinct from the pattern found in glucose-containing culture, indicating that the cellulolytic genes are regulated by SsCebR (2). Furthermore, the xylan-specific secretion of multiple xylanases, esterases, and pectate lyases was reported (2), although transcriptional regulation of genes encoding those enzymes in cellulolytic *Streptomyces* is currently elusive, and intracellular proteomic responses to xylan are also not reported.

In this study, we performed extracellular proteomics and pair-wise Tandem Mass Tag (TMT)-labeling quantitative intracellular proteomics of SirexAA-E grown on either glucose-, xylose-, xylobiose-, or xylan-containing medium to gain knowledge of both extracellular and intracellular responses of SirexAA-E. Furthermore, pull-down proteomics was conducted to elucidate possible xylose and xylan-responsive transcriptional regulators. The extent of the xylan-specific metabolic circuitry in SirexAA-E revealed by the intracellular proteomes has given new insights into the robust metabolic capability of a cellulolytic *Streptomyces*.

## RESULTS

### Growth of SirexAA-E on xylose, xylobiose, and xylan

To test the effect of different carbon sources on the growth of SirexAA-E, the cells were grown in the presence of xylose, xylobiose, and xylan as sole carbon sources along with the cells grown on glucose as a control. The cell growths were monitored by the real-time PCR targeting rRNA DNAs in the SirexAA-E genome (Fig. S1) at 24, 48, 72, and 120 h. SirexAA-E grew well on all tested carbon sources and entered the early stationary phase at around 72 h (2, 8). From this result, the SirexAA-E cells grown on different carbon sources were cultivated at 72 h for the following experiments.

### Xylose, xylobiose, and xylan-specific extracellular proteomes

**Fig 1:**
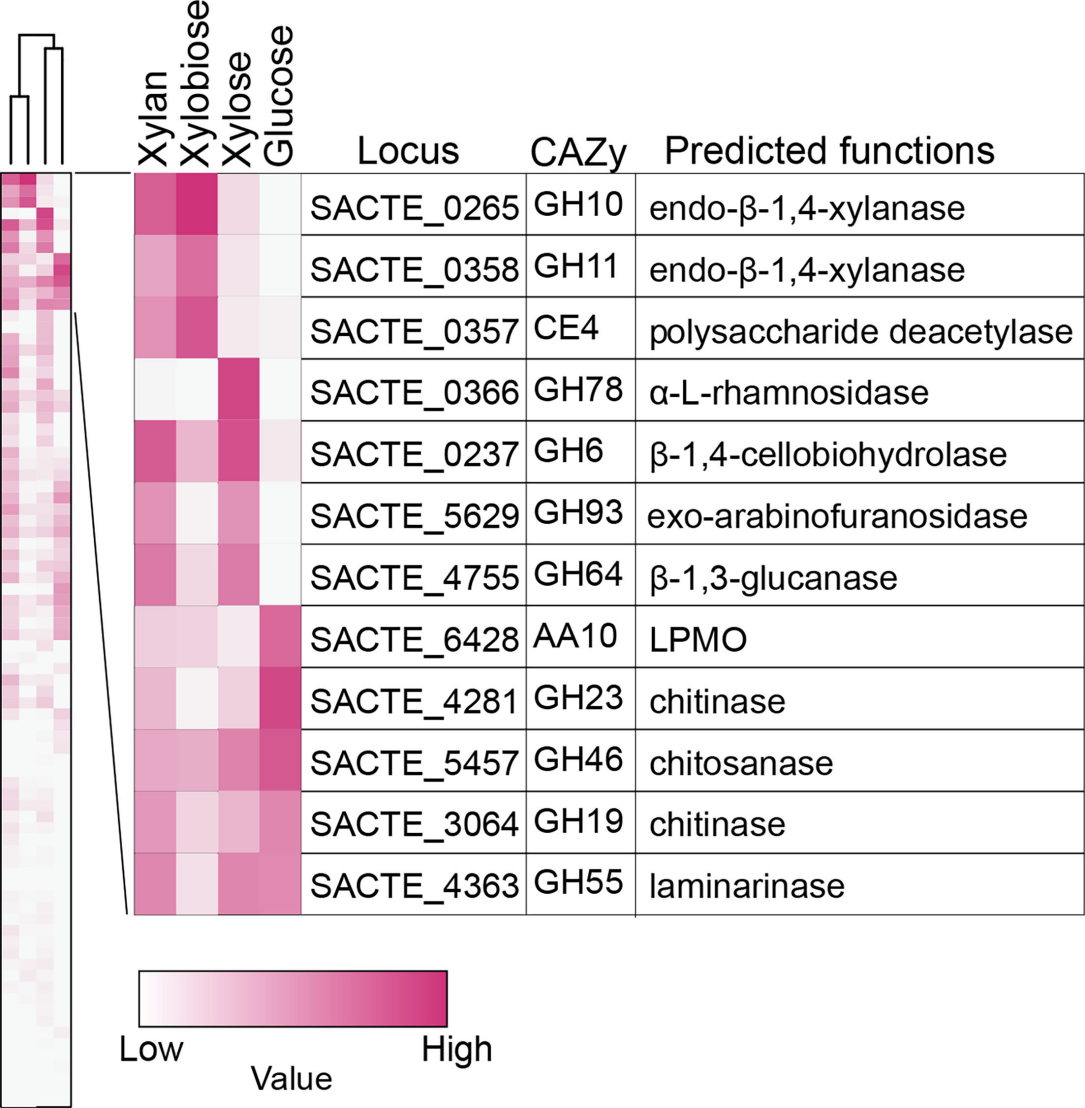
A heatmap showing expression levels of the extracellular CAZymes in the culture supernatant from the cells grown on xylose, xylobiose, xylan, and glucose. A hierarchical grouping is shown based on the secretion pattern in each sample. The carbon source-specific CAZyme secretions are shown. The protein abundance is color-coded as indicated.

Earlier, we showed SirexAA-E secretes a set of xylan-degrading enzymes in response to xylan, though the observed response was not studied in sufficient detail to assess whether the secretion was triggered by a monosaccharide xylose, disaccharide xylobiose, or longer xylooligosaccharides in the xylan hydrolysates (2). To assess whether SirexAA-E responds to growth on either xylose, xylobiose, or xylan by secreting different sets of enzymes, extracellular proteomics analyses were performed and compared to a glucose-grown control (Tables S1 through S4 and PRIDE database: PXD061680). Figure1 shows a hierarchical order of extracellular CAZymes associated with growth on either xylose, xylobiose, xylan, or glucose. When xylan was used as a sole carbon source in the medium, xylan-degrading enzymes such as the endo-β−1,4-xylanases SACTE_0265 (GH10) and SACTE_0358 (GH11), polysaccharide deacetylase SACTE_0357 (CE4), and exo-arabinofuranosidase SACTE_5629 (GH93) were highly secreted together with β−1,4-cellobiohydrolase SACTE_0237 (GH6) and β−1,3-glucanase SACTE_4755 (GH64), consistent with the previous study (2). In the xylobiose-grown extracellular proteome (Fig. 1), the overall composition was like that of the xylan-grown extracellular proteome, with significant enrichment of two endo-β−1,4-xylanases SACTE_0265 (GH10) and SACTE_0358 (GH11), polysaccharide deacetylase SACTE_0357 (CE4), and less abundant SACTE_5629 (GH93) and SACTE_4755 (GH64). In the xylose-grown extracellular proteome, α-L-rhamnosidase SACTE_0366 (GH78) was uniquely secreted, and this enzyme may hydrolyze α-linked L-rhamnosides from the polysaccharide component of pectin in the plant cell wall (9).

Three predicted chitin-degrading enzymes, namely, SACTE_3064 (GH19), SACTE_4281 (GH23), SACTE_5457 (GH46); one LPMO (SACTE_6428) (AA10); and biochemically characterized GH55 laminarinase (SACTE_4363) were detected in the glucose culture supernatant. These extracellular enzymes are not specifically induced by either xylan or other xylan derivatives (7). From these results, it appears that most secreted xylan-utilizing enzymes are induced by xylobiose and xylose, which are the two major soluble products in the xylan hydrolysate.

### COG assignments from intracellular quantitative proteomics

To gain insights into how the intracellular proteome of SirexAA-E changes in response to pentose compounds (i.e., xylose and xylobiose) relative to hexose compounds (i.e., glucose), and as a comparison to the extracellular proteome, a pairwise TMT-labeling intracellular proteomics was performed in two biological replicates for xylose/glucose, xylobiose/glucose, and xylan/glucose samples (Fig. S2 and PRIDE database: PXD067972). The correlation coefficients between two datasets in xylose/glucose, xylobiose/glucose, and xylan/glucose were *R* = 0.75 (*P* value < 0.0001), *R* = 0.69 (*P* value < 0.0001), and *R* = 0.76 (*P* value < 0.0001), respectively, indicating that they are highly correlated. Therefore, the data set with a higher number of detected proteins was used for the subsequent analyses. There were 1,530, 1,354, and 1,564 proteins determined in the proteome data sets of xylose/glucose, xylobiose/glucose, and xylan/glucose, respectively (Fig. S3 and Tables S5 through S7), and 1,037 proteins were found in all data sets (Table S8), which enabled us to compare their intracellular proteomes in a quantitative manner. In the genome of SirexAA-E, there are 6,562 protein-coding genes; thus, >20% of total gene products were identified to undergo changes between pentose and hexose metabolism.

**Fig 2:**
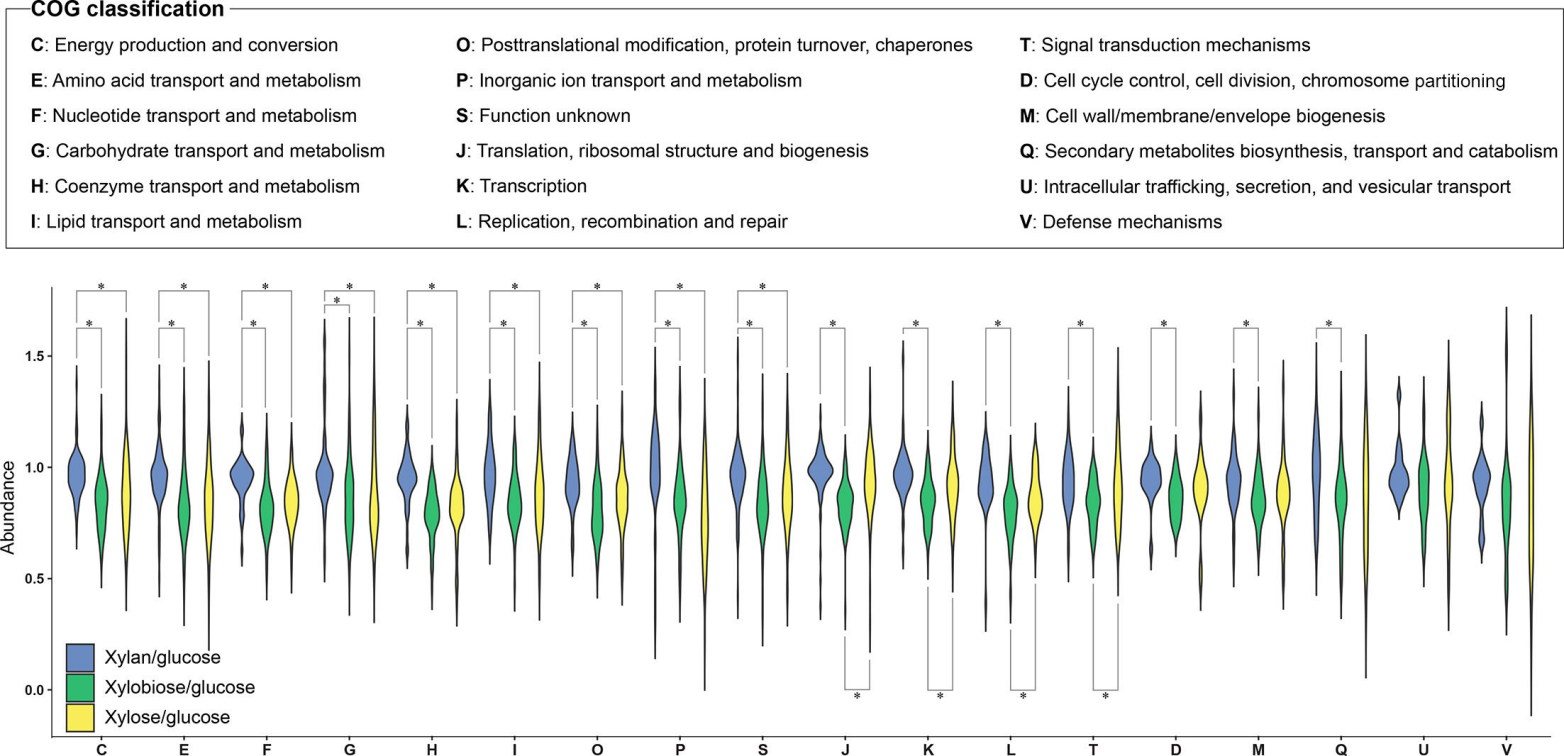
A COG classification based on quantitative intracellular proteomics analysis. Quantified 1,037 proteins in all three proteomic data sets (xylan/glucose; blue, xylobiose/glucose; green, and xylose/glucose; yellow) were sorted into 18 COG categories and analyzed by violin plots. The brackets with asterisks show statistically significant differences between the two datasets estimated by the Student’s *t*-test (*P*-value < 0.05).

There were 221, 78, and 216 proteins uniquely unique to the xylose/glucose, xylobiose/glucose, and xylan/glucose data sets, respectively (Fig. S3 and Table S8). Furthermore, there were 100, 139, and 172 proteins shared between the xylose/glucose and xylobiose/glucose, xylobiose/glucose and xylan/glucose, and xylose/glucose and xylan/glucose data sets, respectively (Fig. S3 and Table S8). The 1,037 proteins present in all data sets were quantified, and the positive Spearman correlations between the abundances of xylan/glucose and xylobiose/glucose (R = 0.54) and xylobiose/glucose and xylose/glucose (R = 0.60) were determined with *p-*values < 2.2e^−16^ (Fig. S4). The 1,037 ubiquitous proteins were categorized into clusters of orthologous genes (COGs) (10) and analyzed using a violin plot (Fig. 2). Statistically significant differences between xylose/glucose and xylan/glucose and xylobiose/glucose and xylan/glucose were observed in the following COG categories: energy production and conversion (COG: C, *n* = 92); amino acid transport and metabolism (COG: E, *n* = 119); nucleotide transport and metabolism (COG: F, *n* = 47); carbohydrate transport and metabolism (COG: G, *n* = 74); coenzyme transport and metabolism (COG: H, *n* = 57); lipid transport and metabolism (COG: I, *n* = 63); posttranslational modification, protein turnover, chaperones (COG: O, *n* = 48); inorganic ion transport and metabolism (COG: P, *n* = 46); and function unknown (COG: S, *n* = 103). Spearman’s correlation analysis between the xylose/glucose and xylobiose/glucose data sets showed a statistically significant positive correlation in nine categories (COGs: C, E, F, G, H, I, O, P, and S) (Fig. S5). Thus, intracellular proteins in these nine categories show attributes of specific regulation by xylan and its major hydrolysis products.

Four categories showed xylobiose-specific regulation: translation, ribosomal structure, and biogenesis (COG: J, *n* = 106); transcription (COG: K, *n* = 60); replication, recombination, and repair (COG: L, *n* = 34); signal transduction mechanisms (COG: T, *n* = 57) (Fig. 2). There was a weak Spearman’s positive correlation between xylan/glucose and xylose/glucose data sets in these four categories (Fig. S6).

The intracellular proteomes assigned in the xylan/glucose and xylobiose/glucose data sets were significantly different in three categories (Fig. 2): cell cycle control, cell division, and chromosome partitioning (COG: D, *n* = 20); cell wall/membrane/envelope biogenesis (COG: M, *n* = 42); secondary metabolites biosynthesis, transport, and catabolism (COG: Q, *n* = 49). A weak correlation of xylan/glucose between xylose/glucose and xylobiose/glucose between xylose/glucose was observed in the D, M, and Q categories (Fig. S7). Positive correlations were determined in the other categories, including U and V (Fig. S8).

From the above results, there is a xylan-specific aspect to intracellular protein expression, possibly driven by the transport of longer xylooligosaccharides such as xylotriose, xylotetraose, or other xylan derivatives.

### Most strongly induced intracellular proteins across all proteomes

**Fig 3:**
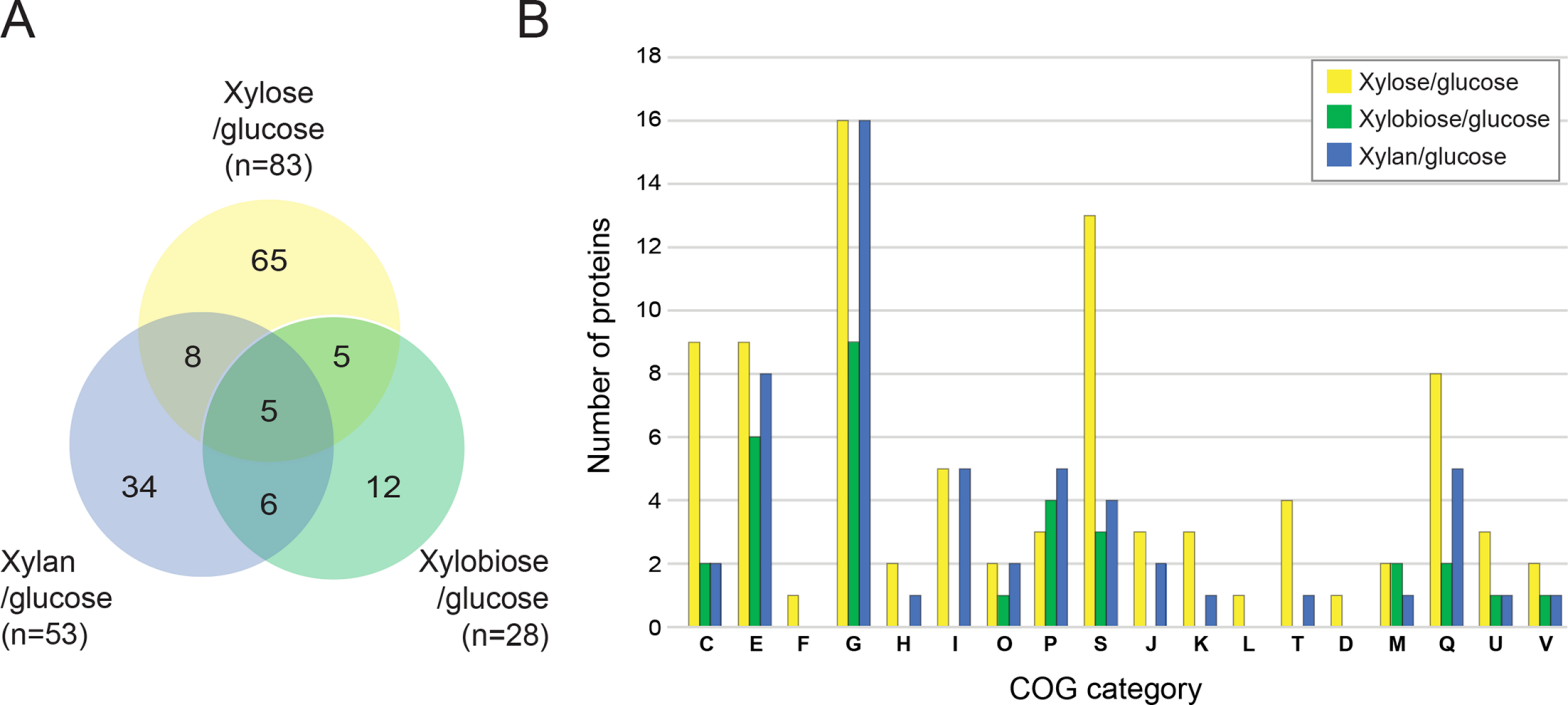
Overlap in the number of intracellular proteins induced by growth on xylose, xylobiose, and xylan. The number of abundant intracellular proteins (>1.5 fold) in xylose/glucose, xylobiose/glucose, and xylan/glucose intracellular proteome data sets is shown as a Venn diagram (**A**) and COG classification (**B**), respectively. The total number of proteins with their corresponding COGs in xylose/glucose (yellow), xylobiose/glucose (green), and xylan/glucose (blue) data sets is shown.

To further examine the intracellular proteins most strongly induced by xylose, xylobiose, or xylan, we chose those with higher than 1.5-fold abundance over the glucose intracellular proteomes (Fig. 3 and Tables 1 and 2). There were 83 xylose/glucose, 28 xylobiose/glucose, and 53 xylan/glucose proteins that exceed this enrichment threshold (Fig. 3). Five proteins were found in all data sets, namely, a xylose isomerase (COG: G; SACTE_5230), xylobiose transporter (COG: G; SACTE_0534), protein or peptide transporters (COG: MU; SACTE_2103 and COG: E; SACTE_4343), and urocanate hydratase (COG: E; SACTE_2533) (Table 2). SACTE_5230 and SACTE_0534 are likely to play essential functions in xylose isomerization and xylooligosaccharide uptake, respectively, showing 83% and 49% identity with their homologs in the well-studied *Streptomyces coelicolor* (11, 12). The peptide transporters presumably enable the secretion of enzymes found in the xylose, xylobiose, and xylan secretomes (Fig. 1). Additionally, a putative urocanate hydratase (SACTE_2533) is thought to catalyze the conversion of urocanate to 4-imidazolone-5-propanoate in histidine metabolism, as detailed later (Fig. S9).

**TABLE 1:** Xylose-, xylobiose-, or xylan-specifically abundant intracellular proteins^*a*^

Xylose-specific	Category	Fold
SACTE_3882 NADH dehydrogenase subunit F	C	4.03
SACTE_3881 NADH-quinone oxidoreductase subunit E	C	3.88
SACTE_5567 cytochrome P450	Q	3.05
SACTE_5760 ribose transport system ATP-binding protein	G	2.94
SACTE_5472 malate synthase	C	2.91
SACTE_3885 NADH-quinone oxidoreductase subunit I	C	2.77
SACTE_1901 alpha-N-arabinofuranosidase	G	2.74
SACTE_0718 peptide/nickel transport system substrate-binding protein	E	2.58
SACTE_6519 NitT/TauT family transport system permease protein	P	2.41
SACTE_1504 transcriptional regulator, TraR/DksA family	T	2.38
SACTE_1900 putative multiple sugar transport system permease protein	G	2.34
SACTE_1707 Bifunctional DNA primase/polymerase, N-terminal	S	2.31
SACTE_2198 dCTP deaminase	F	2.19
SACTE_3888 NADH-quinone oxidoreductase subunit L	CP	2.09
SACTE_3849 Protein of unknown function (DUF2809)	S	2.03
SACTE_2094 von Willebrand factor type A domain-containing protein	T	2.02
SACTE_4368 ATP-binding protein	–^*b*^	1.99
SACTE_6380 tRNA threonylcarbamoyl adenosine modification protein	J	1.93
SACTE_3412 fatty-acyl-CoA synthase	IQ	1.86
SACTE_5820 glycine betaine/proline transport system permease protein	E	1.84
SACTE_5566 cytochrome P450	Q	1.83
SACTE_1974 small subunit ribosomal protein S20	J	1.82
SACTE_5579 acyl transferase domain-containing protein	Q	1.81
SACTE_1934 pyruvate phosphate dikinase	G	1.81
SACTE_1899 monosaccharide ABC transporter ATP-binding protein	G	1.79
SACTE_3883 NADH-quinone oxidoreductase subunit G	C	1.78
SACTE_3179 cold-shock DNA-binding protein family	K	1.77
SACTE_1896 2-desacetyl-2-hydroxyethyl bacteriochlorophyllide A dehydrogenase	E	1.77
SACTE_5585 putative ABC transport system permease protein	V	1.76
SACTE_5571 amino acid adenylation domain-containing protein	Q	1.74
SACTE_5763 D-ribose pyranase	G	1.72
SACTE_3160 cold-shock DNA-binding protein family	K	1.67
SACTE_0914 peptidyl-prolyl cis-trans isomerase B (cyclophilin B)	O	1.67
SACTE_1063 sec-independent protein translocase protein TatA	U	1.66
SACTE_4569 F-type H+-transporting ATPase subunit epsilon	C	1.66
SACTE_0368 multiple sugar transport system substrate-binding protein	G	1.63
SACTE_2427 protein of unknown function (DUF3039)	S	1.62
SACTE_3832 uncharacterized protein, contains SIS phosphosugar binding domain	S	1.62
SACTE_3999 ATPase components of ABC transporters with duplicated ATPase domains	S	1.62
SACTE_5983 glyceraldehyde 3-phosphate dehydrogenase	G	1.61
SACTE_0313 Protein of unknown function (DUF2795)	S	1.61
SACTE_3692 S-adenosyl methyltransferase	S	1.61
SACTE_4775 DNA-binding protein HU-beta	L	1.60
SACTE_5740 monosaccharide ABC transporter substrate-binding protein, CUT2 family	G	1.59
SACTE_4224 phosphoenolpyruvate carboxykinase (GTP)	H	1.59
SACTE_1250 serine protease, subtilisin family	O	1.58
SACTE_5904 ATPase components of ABC transporters with duplicated ATPase domains	S	1.58
SACTE_5759 transcriptional regulator, LacI family	K	1.57
SACTE_2610 ATP-binding cassette, subfamily F, uup	S	1.57
SACTE_1267 ATPase components of ABC transporters with duplicated ATPase domains	S	1.56
SACTE_1140 Acyl dehydratase	I	1.56
SACTE_1020 D-serine deaminase, pyridoxal phosphate-dependent	E	1.56
SACTE_5804 tetratricopeptide repeat-containing protein	D	1.55
SACTE_3890 NADH-quinone oxidoreductase subunit N	C	1.55
SACTE_0919 preprotein translocase subunit SecD	U	1.55
SACTE_2745 signal transduction histidine kinase	T	1.54
SACTE_5394 pyruvate dehydrogenase (quinone)	EH	1.54
SACTE_1895 galactonate dehydratase	M	1.53
SACTE_4639 Zn-dependent metalloprotease	E	1.53
SACTE_6222 L-histidine Nalpha-methyltransferase	S	1.52
SACTE_2195 polyketide cyclase / dehydrase and lipid transport	I	1.52
SACTE_0720 3-oxoacyl-[acyl-carrier protein] reductase	IQ	1.52
SACTE_5569 [acyl-carrier-protein] S-malonyltransferase	I	1.52
SACTE_5581 acyl transferase domain-containing protein	Q	1.51
SACTE_4359 ABC-2 type transport system ATP-binding protein	V	1.51

Xylobiose-specific	Category	Fold
SACTE_1689 oleandomycin transport system ATP-binding protein	V	3.12
SACTE_3884 NADH-quinone oxidoreductase subunit H	C	2.41
SACTE_2174 5-aminolevulinate synthase	E	2.28
SACTE_0358 endo-1,4-beta-xylanase	G	2.24
SACTE_2561 SurA N-terminal domain-containing protein	O	2.03
SACTE_4344 oligopeptide transport system permease protein	EP	1.90
SACTE_6450 L-rhamnose isomerase / sugar isomerase	M	1.87
SACTE_4708 glucose/arabinose dehydrogenase, beta-propeller fold	G	1.87
SACTE_2454 alpha-glucoside transport system substrate-binding protein	G	1.64
SACTE_0636 iron complex transport system permease protein	P	1.57
SACTE_5493 simple sugar transport system substrate-binding protein	G	1.53
SACTE_1469 TIGR03085 family protein	C	1.51

Xylan-specific	Category	Fold
SACTE_2289 cellobiose-binding protein	G	3.52
SACTE_0882 DNA-directed RNA polymerase subunit omega	K	2.89
SACTE_1650 maltooligosaccharide transport system substrate-binding protein	G	2.74
SACTE_0400 sorbitol/mannitol transport system substrate-binding protein	G	2.53
SACTE_5227 multiple sugar transport system permease protein	G	2.37
SACTE_2711 Na+/H+-dicarboxylate symporter	C	2.19
SACTE_5229 multiple sugar transport system substrate-binding protein	G	2.18
SACTE_4493 nickel superoxide dismutase	S	2.07
SACTE_0532 xylobiose transport system permease protein	G	2.06
SACTE_1522 carbonic anhydrase	P	2.03
SACTE_2286 beta-glucosidase	G	2.02
SACTE_5228 multiple sugar transport system permease protein	G	1.99
SACTE_2739 methylglutaconyl-CoA hydratase	I	1.93
SACTE_4991 conserved hypothetical protein	–	1.91
SACTE_4284 peroxiredoxin (alkyl hydroperoxide reductase subunit C)	O	1.87
SACTE_3685 carbohydrate ABC transporter ATP-binding protein, CUT1 family	E	1.87
SACTE_2494 hypothetical protein	S	1.86
SACTE_1889 3-oxoacyl-[acyl-carrier-protein] synthase II	IQ	1.74
SACTE_4757 acetyl-CoA carboxylase carboxyltransferase subunit	I	1.66
SACTE_1886 [acyl-carrier-protein] S-malonyltransferase	I	1.66
SACTE_3271 DnaJ domain	O	1.66
SACTE_1239 enoyl-[acyl-carrier-protein] reductase [NADH]	I	1.65
SACTE_1597 conserved hypothetical protein	–	1.65
SACTE_5334 NitT/TauT family transport system ATP-binding protein	P	1.64
SACTE_2470 conserved hypothetical protein	–	1.62
SACTE_6209 S-adenosyl methyltransferase	J	1.58
SACTE_1903 hypothetical protein	S	1.57
SACTE_6309 iron complex transport system permease protein	P	1.56
SACTE_4191 histidine ammonia-lyase	E	1.53
SACTE_5584 putative ABC transport system ATP-binding protein	V	1.53
SACTE_6445 LPXTG-motif cell wall anchor domain-containing protein	–	1.53
SACTE_3601 thiosulfate/3-mercaptopyruvate sulfurtransferase	P	1.52
SACTE_0397 2-desacetyl-2-hydroxyethyl bacteriochlorophyllide A dehydrogenase	E	1.51
SACTE_6304 isochorismate synthase	HQ	1.50

^*a*^ Intracellular proteins detected in only one of three datasets: xylose/glucose, xylobiose/glucose, or xylan/glucose, showing >1.5 fold increase in the abundance relative to the glucose intracellular proteome, is shown.

^*b*^ –, proteins are not assigned to any COG category.

**TABLE 2:** >1.5-fold enriched proteins determined in four groups of the Venn diagram in Fig. 3A^*a*^

Xylan and xylobiose	Category^*b*^	Xylan	Xylobiose	
SACTE_4283 alkyl hydroperoxide reductase subunit D	S	2.88	1.82	
SACTE_0529 Na+/proline symporter	E	2.22	2.09	
SACTE_5190 drug resistance transporter, EmrB/QacA subfamily	EGP	2.18	1.66	
SACTE_0638 L-ornithine N5-oxygenase	Q	2.07	1.50	
SACTE_0531 beta-xylosidase	G	1.95	1.52	
SACTE_2530 imidazolonepropionase	Q	1.52	1.78	

Xylan and xylose	Category	Xylan	Xylose	
SACTE_1898 putative multiple sugar transport system substrate-binding protein	G	2.23	2.67	
SACTE_5231 alpha-D-xyloside xylohydrolase	G	2.02	1.82	
SACTE_5226 beta-glucosidase	G	1.87	1.57	
SACTE_5827 xylulokinase	G	1.66	1.72	
SACTE_2308 polar amino acid transport system substrate-binding protein	ET	1.57	1.92	
SACTE_6141 NADPH:quinone reductase	C	1.56	2.02	
SACTE_3309 LSU ribosomal protein L34P	J	1.52	1.79	
SACTE_1287 ectoine hydroxylase	Q	1.51	1.53	

Xylobiose and xylose	Category	Xylobiose	Xylose	
SACTE_4612 putative cholesterol esterase	–^*b*^	2.04	1.51	
SACTE_5761 ribose transport system permease protein	G	1.99	2.09	
SACTE_0953 sugar phosphate permease	P	1.83	2.58	
SACTE_0009 protein of unknown function (Hypoth_ymh)	S	1.78	2.33	
SACTE_1248 cation/acetate symporter	S	1.61	1.76	

Found in all	Category	Xylose	Xylobiose	Xylan
SACTE_5230 xylose isomerase	G	2.29	2.98	3.54
SACTE_0534 xylobiose transport system substrate-binding protein	G	2.12	3.04	3.64
SACTE_2103 LPXTG-motif cell wall anchor domain-containing protein	MU	2.13	1.61	1.96
SACTE_4343 oligopeptide transport system substrate-binding protein	E	1.83	1.78	1.66
SACTE_2533 urocanate hydratase	E	1.78	1.63	1.56

^*a*^ Intracellular proteins found in the following datasets, xylose/glucose and xylobiose/glucose, xylobiose/glucose and xylan/glucose, xylose/glucose and xylan/glucose, and xylose/glucose, xylobiose/glucose and xylan/glucose, with >1.5 fold increase in abundance relative to the glucose intracellular proteome, corresponding to the Venn diagram shown in Fig. 3A.

^*b*^ –, proteins are not assigned to any COG category.

A total of 65, 12, and 34 proteins were uniquely and highly expressed in the xylose/glucose, xylobiose/glucose, and xylan/glucose data sets, respectively (Fig. 3A and Table 1). The 65 highly expressed proteins in the xylose/glucose data set include seven proteins in the “energy production and conversion” (COG: C), nine in “carbohydrate transport and metabolism” (COG: G), seven in COG: Q, ten in COG: S, and others. Among the COG: C proteins, SACTE_5472 is a predicted malate synthase (MS), and the rest were annotated with a role in oxidative phosphorylation, i.e., generation of ATP.

### Xylose- and xylobiose-associated intracellular proteins

Among the xylose-related proteins, 16 proteins were annotated as either an ATP-binding protein or subunit of an ABC transporter across four COG categories (COG: G, E, S, and V), which might support xylose uptake by using ATP hydrolysis as a driving force (Tables 1 and 2). Additionally, a fatty-acyl-CoA synthase (COG: IQ; SACTE_3412), enoyl-CoA hydratase (COG: I; SACTE_1140), acyl-carrier-protein S-malonyltransferase (COG: I; SACTE_5569), and acyl transferase domain-containing protein (COG: Q; SACTE_5581) were potentially involved in β-oxidation to produce acetyl-CoA (13), although most β-oxidation enzymes were more abundant in the glucose intracellular proteome (Tables S6 through S8). There were two predicted translocases (COG: U; SACTE_0919 and SACTE_1063) (14, 15), and hence they may support the secretion of α-L-rhamnosidase and other xylose-specific enzymes found in the xylose secretome (Fig. 1).

The 12 xylobiose-specific highly expressed proteins included four proteins categorized in COG: G, two COG: C, and others (Fig. 3B and Table 1). COG: G contains predicted endo-β−1,4-xylanase (SACTE_0358) (GH11), two ABC transporters (SCTE_2454 and SACTE_5493), and glucose or arabinose dehydrogenase (SACTE_4708). The endo-β−1,4-xylanase (SACTE_0358) was also found in the culture supernatant when cells were grown in xylobiose (Fig. 1). SACTE_2454 and SACTE_5493 (COG: G) and SACTE_1689 (COG: V) are thought to transport xylobiose into the cells in an ATP-dependent manner (16). Among the xylobiose-specific highly expressed proteins, SACTE_3884 (COG: C) was the only protein that may function in oxidative phosphorylation for ATP synthesis.

Five upregulated proteins were shared by xylose/glucose and xylobiose/glucose intracellular proteome data sets, including three transporters: SACTE_0953 (COG: P), SACTE_1248 (COG: S), and SACTE_5761 (COG: G); one putative cholesterol esterase; and one with unknown function. These transporters are thought to be xylose and xylobiose responsive transporters. Eight upregulated proteins were found in both xylose/glucose and xylan/glucose data sets, including three predicted xylanase and xylose utilization enzymes (COG: G; SACTE_5226, SACTE_5231, and SACTE_5827), a sugar transporter (COG: G; SACTE_1898), a polar amino acid transporter (COG: E; SACTE_2308), NADPH:quinone reductase in the oxidative phosphorylation pathway (COG: C; SACTE_6141), and two others (COG: Q; SACTE_1287, COG: J; SACTE_3309). Six proteins were found in both xylobiose/glucose and xylan/glucose data sets, including a putative xylosidase that may produce xylose from xylooligosaccharide (COG: G; SACTE_0531), an Na^+^/proline symporter (COG: E; SACTE_0529), a drug-resistant transporter (COG: EGP; SACTE_5190), and three others (COG: S; SACTE_4283, COG: Q; SACTE_0638 and SACTE_2530) (Fig. 3 and Table 1).

### Xylan-associated intracellular proteins, including transporters

There were 34 intracellular proteins uniquely expressed during growth on xylan, including one protein functioning in the oxidative phosphorylation pathway (COG: C). Eight proteins in the COG: G included a predicted xylobiose transport system permease protein (SACTE_0532), β-glucosidase (SACTE_2286) (GH1), cellobiose-binding protein (SACTE_2289), and multiple sugar transport system substrate-binding protein (SACTE_5229) (Table 1). Indeed, SACTE_0532 has a close homolog in xylulolytic *S. thermoviolaceus* OPC-520 (protein_ID), which is biochemically characterized as a xylobiose ABC transporter (16).

### Determination of ATP/ADP balance

**Fig 4:**
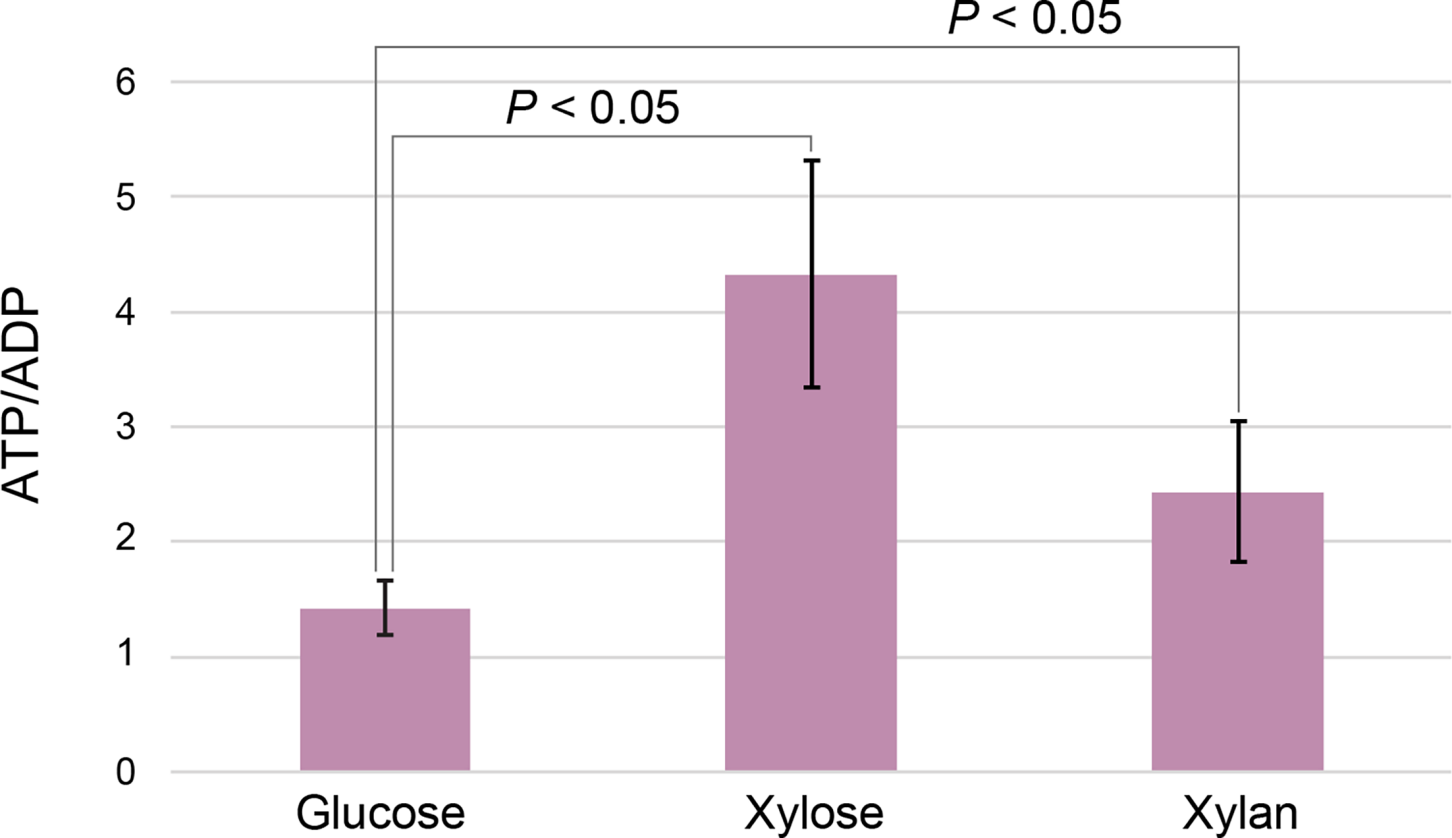
ATP/ADP ratio in SirexAA-E grown on xylose, xylan, or glucose after 3 days of cultivation. Data represent mean ± SD from three biological replicas (*N* = 3). *P-*values less than 0.05 are shown between samples.

When SirexAA-E was grown on xylose, xylobiose, and xylan, significantly more enzymes related to oxidative phosphorylation and ABC transportation were expressed than in glucose-grown cells. Thus, the ratio between intracellular ATP and ADP was determined in the cells grown on glucose, xylose, or xylan (Fig. 4). When the cells were grown on xylose or xylan, ~4.4 and 2.5 excess of ATP compared to ADP was determined, which was significantly different from the ATP/ADP ratio of ~1.5 in the glucose-grown cells. Thus, in the presence of xylose or xylan, the expression of oxidative phosphorylation genes corresponds with an increased supply of ATP to support energy-consuming processes.

### Glutamate production from histidine and 2-oxoglutarate

Bacteria can accumulate glutamate as a pool of nitrogen source for production of other amino acids (17, 18). Consistently, we observed that certain pathways leading to glutamate accumulation were activated, as mapped by both mRNA abundance (2) and intracellular abundance (Fig. S1). Among enzymes from these pathways, SACTE_2530 is a predicted imidazolone propionase, which promotes the conversion of histidine into glutamate together with the urocanate hydratase (COG: E; SACTE_2533, Fig. S9 and S10 and Tables S5 through S7). From xylulose-5P, 11 steps are needed to synthesize histidine, and the three following reactions from histidine to glutamate are catalyzed by SACTE_4191, SACTE_2533, and SACTE_2530. The latter three enzymes were upregulated in the xylose/glucose, xylobiose/glucose and xylan/glucose intracellular proteome data sets (Fig. S9). These observations suggest that the catabolism of histidine to glutamate is needed when the cells are grown on xylose, xylobiose, or xylan.

Oxoglutarate dehydrogenase (SACTE_4509, OGDH) and succinyl-CoA synthetase (SACTE_4072 and 4073, SCS) showed low transcript abundance and low protein accumulation, suggesting restriction of flux in this step of the TCA cycle, while production of glutamate from 2-oxoglutarate could be catalyzed by two glutamate synthases SACTE_1449 and SACTE_1450, and two glutamate dehydrogenases, SACTE_2468 and SACTE_4198 (Fig. S10), which along with glutamate dehydrogenase (SACTE_2468, GH), were readily abundant in the xylobiose/glucose and xylan/glucose data sets. These results support the requirement for additional amino acids to synthesize secreted proteins involved in xylan catabolism when compared to the cells grown on glucose.

### Conserved sugar transporters in several genomic regions

**Fig 5:**
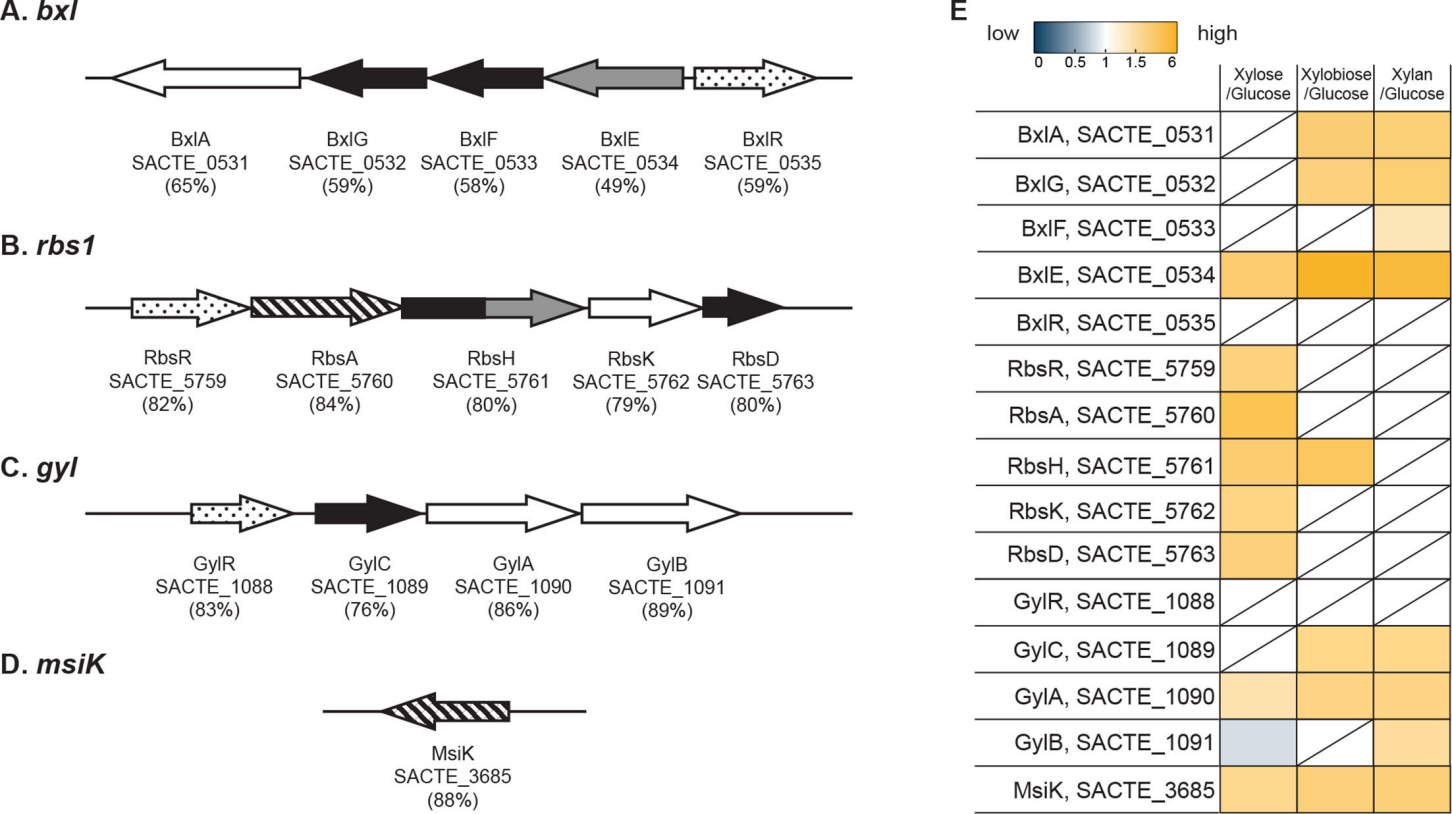
Putative sugar transporter genes identified among the quantified SirexAA-E proteome. The percentage indicates protein identity to those in the *S. coelicolor* genome. For (**A through D**), genes that encode membrane proteins are shown in black, and genes that encode substrate-binding proteins are shown in gray, regulatory genes are shown as dotted arrows, and ATP-binding and/or hydrolyzing genes are indicated as arrows with diagonal stripes. (**A**) *bxl* system includes *bxlA*, *bxlG*, *bxlF, bxlE,* and *bxlR* for a putative xylobiose utilization via the ABC transportation system. (**B**) *rbs1* system includes *rbsR*, *rbsA*, *rbsH*, *rbsK,* and *rbsD* for ribose utilization via the ABC transportation system. (**C**) *gyl* system includes *gylR*, *gylC*, *gylA,* and *gylB* for glycerol utilization by facilitated diffusion. (**D**) *msiK* encodes an ATPase that functions as a ubiquitous ABC transport system. (**E**) Protein abundance of the depicted proteins from the quantitative intracellular proteomics according to the color code.

Several representative gene clusters for sugar transporters (i.e., ABC transporter, PTS, and others) were reported in *S. coelicolor* (11, 19–22). Homologs of these gene clusters were found in Sirex AA-E with few alterations such as the order or direction of genes in the cluster. Out of 13 conserved putative carbohydrate transport-related gene clusters in the SirexAA-E genome, three transporter clusters and ATPase subunit (MsiK) showed xylose-, xylobiose-, or xylan-dependent regulation. They include genes encoding for a potential xylooligosaccharide permease BxlEFG, ribose permease RbsADHK, glycerol permease GylCAB, and MsiK for ABC transporters (Fig. 5A through D) (11).

The *bxl1* cluster was assigned to xylooligosaccharide uptake in *S. coelicolor* and was conserved in the SirexAA-E genome, including BxlEFG permease genes (SCTE_0532-SACTE_0534), BxlA β-D-xylosidase gene (SACTE_0531), and BxlR LacI family transcriptional regulator (SACTE_0535) (Fig. 5A). The proteins encoded by the *bxl1* cluster were abundantly expressed in the xylobiose/glucose and xylan/glucose proteome data sets, except that BxlF (SACTE_0533) was not detected in the xylobiose/glucose data set. Moreover, except for BxlE (SACTE_0534), the cluster was missing in the xylose/glucose data set, suggesting that the *bxl1* cluster in SirexAA-E is regulated in a more specific xylobiose- and xylan-dependent manner (Fig. 5E). SACTE_0535 was not identified in any data set, which might be due to the absence of this regulator in either the glucose-grown cells or xylose, xylobiose, and xylan-grown cells. To note, there are two *bxl* clusters, *bxl1* and *bxl2*, in the *S. coelicolor* genome, while only *bxl1* was found in the SirexAA-E genome.

Three possible ABC transporter systems, encoded in the *rbs1*, *rbs2*, and *rbs3* clusters, for ribose uptake were identified in the SirexAA-E genome, but only proteins encoded in the *rbs1* cluster were detected (Fig. 5B), including a LacI family RbsR (SACTE_5759) and RbsAHKD (SACTE_5760, SACTE_5761, SACTE_5762, and SACTE_5763). With the exception of RbsH, which was found in the xylobiose/glucose data set, more proteins were observed only in the xylose/glucose data set, clearly showing xylose-dependent regulation of the *rbs1* cluster.

A putative *gyl* cluster was identified in the SirexAA-E genome and gene products for GylR (SACTE_1088), GylC (SACTE_1089), GylA (SACTE_1090), and GylB (SACTE_1091) are well conserved with those in the *S. coelicolor*. Except for a LacI-like repressor, GylR not detected in any data set (Fig. 5E), the other *gyl* cluster proteins were abundant in the xylobiose/glucose and xylan/glucose data sets.

MsiK is a predicted ubiquitous ATPase subunit for ABC system, which functions in *trans* and is thus thought to function with multiple ABC uptake systems in *S. coelicolor* (23). The MsiK protein sequence in SirexAA-E is highly conserved ~88% when compared to the sequence in *S. coelicolor*. MsiK was highly enriched in all data sets, indicating an ABC system activated during growth on xylose, xylobiose, and xylan.

### Pull-down proteomics of key transcriptional regulators in response to xylan

To determine transcriptional regulators that upregulate the abovementioned highly enriched proteins, including xylose isomerase, sugar transporters, and others, pull-down proteomics was employed using biotinylated promoter regions of SACTE_0265, 0358, and 5230 (Table 3; Tables S9 through S15). SACTE_0265 and 0358 encode putative endo-β−1,4-xylanases belonging to GH10 and GH11, respectively, both of which were determined to be the two highest secreted enzymes. SACTE_5230 is a highly enriched annotated intracellular xylose isomerase, which is the first enzyme to metabolize xylose inside of the cell. A total of six conditions, comprising the three promoter regions and the two intracellular proteomes, were examined from either glucose- or xylan-grown cells. After excluding transcriptional regulators derived from glucose-grown cells, five new transcriptional regulators from xylan-grown cells were identified. These included LacI-like (SACTE_0535, SACTE_5759), AsnC-like (SACTE_2715), PadR-like (SACTE_3328) and IclR-like (SACTE_5479) regulators, suggesting that these predicted transcriptional regulators specifically recognize upstream regions of targeted xylolytic genes in SirexAA-E.

**TABLE 3:** Transcriptional regulators identified by pull-down that bind to specific promoter regions in cells grown on xylan^*a*^

			Protein abundance (Sequest HT)		
Locus	Description	Aa	P0265	P0358	P5230
SACTE_0535	LacI-like transcriptional regulator	364	13.7	21.6	13.8
SACTE_2715	AsnC-like transcriptional regulator	151	9.8	14.1	12.5
SACTE_3328	PadR-like transcriptional regulator	233	6.5	10.9	12.7
SACTE_5479	IclR-like transcriptional regulator	266	7.6	7.3	4.3
SACTE_5759	LacI-like transcriptional regulator	338	15.0	13.7	6.7

^*a*^ The promoter regions associated with two xylanases and xylose isomerase (P0265, P0358, and P5230) were used to probe intracellular proteins from the xylan- and glucose-grown cultures (Tables S9 through S14). Transcriptional regulators that were found in both cultures were excluded. Score Sequest HT was used as a protein abundance indicator (*n* = 3).

Among the determined putative transcriptional regulators, we sought conserved DNA-binding motifs in the tested promoter regions, and the 5′-CGAANNTTTCG-3′ sequence was found to be a unique and conserved motif in the P0265 and P0358 regions (Fig. S11). Additionally, the same motif was also found in the upstream sequence from the SACTE_0535 gene. The reported homolog of SACTE_0535 in *S. thermoviolaceus*, BxlR, was shown to recognize a similar motif, 5′-CGAANxTTCG-3′; hence, SACTE_0535 is thought to likely function in an analogous way to regulate downstream genes (16).

## DISCUSSION

### Protein secretion by SirexAA-E for xylan degradation

To understand the xylan-specific responses of SirexAA-E with regard to extracellular and intracellular proteome changes, secreted proteins induced by xylose, xylobiose, xylan, and glucose were first compared by the extracellular proteomics (Fig. 1). Pull-down proteomics suggested that the production of secreted enzymes, such as xylan- and xylobiose-specific secretions of xylanases (SACTE_0265 (GH10), 0358 (GH11)) and polysaccharide deacetylase (SACTE_0357 (CE4)), is regulated by one of the transcriptional regulators binding to the promoter regions depending on the presence of xylobiose or xylan (Table 3). A putative α-L-rhamnosidase, SACTE_0366 (GH78), was strongly produced when the cells were grown on xylose as a sole carbon source, corresponding to how the hemicellulose fraction of the plant cell wall might be metabolized in the natural environment. The secretion of cellobiohydrolase SACTE_0237 (GH6), SACTE_5629 (GH93), and SACTE_4755 (GH64) was determined to be induced by xylose and xylan and less so by xylobiose. Notably, SACTE_0237 (GH6) is one of the major cellulases detected in the cellulose culture, and its gene expression was regulated by cellobiose via SsCebR repressor (2, 4), implying that SsCebR may respond to another ligand such as xylose or xylooligosaccharides besides the presently identified cellobiose and mannobiose (2, 4, 8). Overall, these extracellular proteomic results indicate that SirexAA-E produces distinct sets of extracellular proteins in response to xylose, xylobiose, and xylan, with some enzyme production regulated at least by a novel transcriptional regulator, SACTE_0535, encoded in the *bxl* gene cluster (Fig. 7).

### Transporters for carbohydrate uptake regulated by xylose, xylobiose, or xylan

Gene clusters and loci for several carbohydrate transporters were identified in the SirexAA-E genome, such as *bxl*, *rbs1*, *gyl*, and *msiK*. These are conserved in other *Streptomyces* genomes, including the model organism *S. coelicolor* (Fig. 5) (11, 23, 24). Xylobiose and longer xylooligosaccharides are the primary products of extracellular xylan hydrolysis in SirexAA-E (2). Putative xylobiose transporter-coding genes, SACTE_0532, SACTE_0533, SACTE_0534, and xylosidase coding gene, SACTE_0531 (GH3), were highly expressed in the xylan transcriptome, and corresponding proteins were detected in the intracellular proteomes (Fig. 5). Based on the predicted structure of the xylobiose ABC transporter from *S. thermoviolaceus* (16, 25, 26), the solute-binding domain SACTE_0534 of this complex was readily detected by our intracellular proteomics along with inner membrane subunits (SACTE_0532; SACTE_0533). The proteins in this cluster are enriched in the xylobiose/glucose and xylan/glucose intracellular proteome data sets and depleted in the xylose/glucose intracellular proteome, which suggests that this cluster is under the regulation of SACTE_0535, a homolog of the reported BxlR regulator in *S. thermoviolaceus* (16). Furthermore, SACTE_0535 was determined in the pull-down proteomics xylan-grown cells using the promoter regions of xylan-induced proteins, indicating function of this LacI-like regulator in xylan-dependent gene regulation (Fig. 5and 7 and Table 3).

The biochemical function of LacI-like family RbsR encoded in the *rbs1* cluster has not been assessed in any *Streptomyces*, but homologs in other bacteria were described to be repressors that regulate downstream genes involved in ribose uptake (27–30). In the present study, all proteins encoded in the *rbs1* cluster were upregulated when cells were grown on xylose, but not on xylobiose or xylan. Moreover, RbsR (SACTE_5759) in SirexAA-E was detected by the pull-down proteomics when the cell lysates were prepared from xylan grown cells. Thus, it is possible that the *rbs1* cluster in SirexAA-E is involved in uptake of both ribose and xylose.

Since GlyR in the *gly* cluster in *S. coelicolor* and *S. clavuligerus* functions as an inducible repressor with metabolites of glycerol and is subject to glucose inhibition (22, 31), our observation of abundant GlyC (SACTE_1089), GlyA (SACTE_1090), and GlyB (SACTE_1091) in the xylan/glucose data sets can be rationalized by the reported glucose catabolism via GlyR (SACTE_1088) (Fig. 5).

MsiK is thought to be a universal ATPase. It is upregulated in the presence of xylose, xylobiose, and xylan and thus supports the xylose and xylooligosaccharide-dependent ABC transport system. After xylan degradation in the extracellular space, the described transporters are expected to function in xylose and xylooligosaccharide uptake. Further experimental validations are needed to elucidate the different roles of regulators in SirexAA-E in response to xylan, including SACTE_0535 and SACTE_5759 (LacI-like regulators), SACTE_2715 (AsnC-like regulator), SACTE_3328 (PadR-like regulator), and SACTE_5479 (IclR-like regulator).

### Xylan metabolism in SirexAA-E

**Fig 6:**
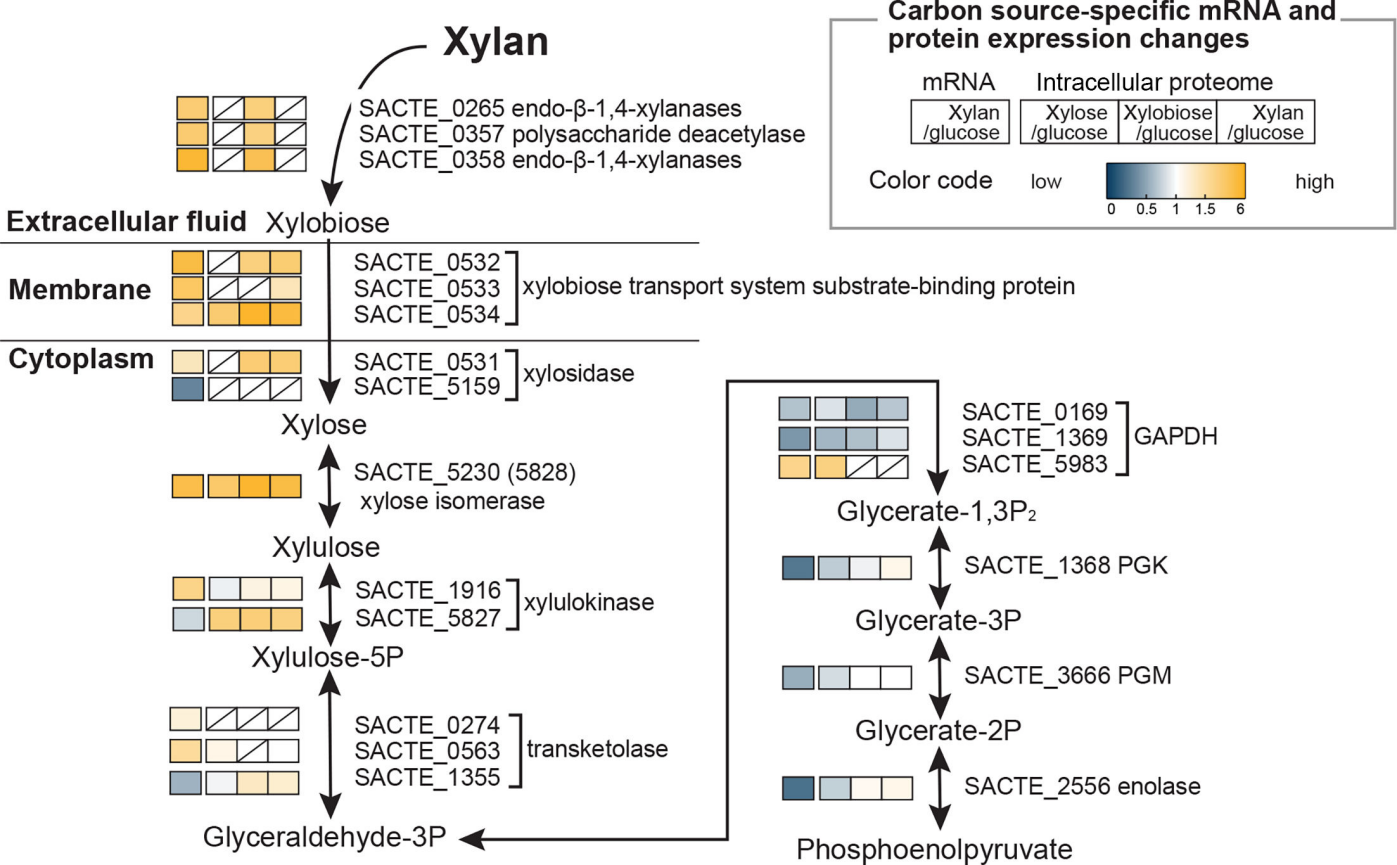
Xylose-, xylobiose-, and xylan-specific responses in a representative metabolic pathway from xylan to phosphoenolpyruvate. The levels of corresponding transcripts (xylan/glucose) from the previous study (2) and enzymes quantified in the present intracellular proteome data sets are shown with color code. The squares with diagonal lines indicate proteins that were not quantified or detected by the proteomics. Abbreviations: GAPDH, glyceraldehyde-3-phosphate dehydrogenase; PGK, phosphoglycerate kinase; PGM, phosphoglycerate mutase.

**Fig 7:**
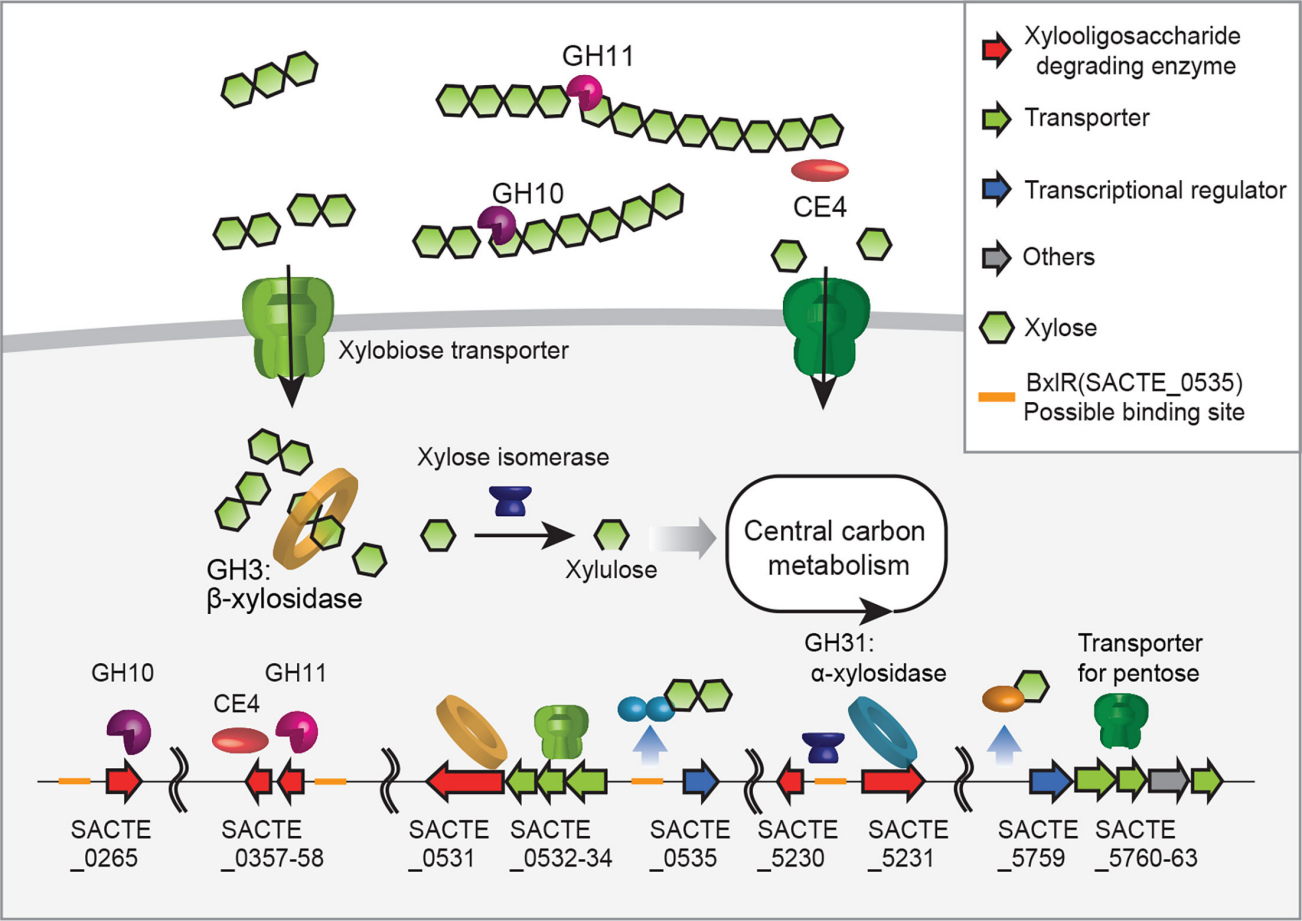
Graphical summary of xylan utilization in SirexAA-E based on our extracellular, intracellular, and pull-down proteomics. Xylan is deacetylated by a deacetylase (CE4: SACTE_0357) and depolymerized by two endo-β−1,4-xylanases (GH10: SACTE_0265 and GH11: SACTE_0358). Xylose is likely uptaken via the putative ABC transporter SACTE_5760–5763, whereas xylobiose and longer xylooligosaccharides are transported through SACTE_0532–0534. The intracellular β-xylosidase (GH3: SACTE_0531) hydrolyzes xylobiose into xylose. Xylose is isomerized into xylulose by a xylose isomerase (SACTE_5230), and xylulose enters central metabolism via the pentose phosphate pathway. Based on the pull-down proteomics, two endo-β−1,4-xylanases (GH10 and GH11) and ABC transporter are thought to be regulated by BxlR (SACTE_0535) via the BxlR-binding site present at their upstream sequences, highlighted in orange. The ABC transporter encoded in the *rbs* cluster (SACTE_5760-5763) possibly uptakes ribose and xylose, and they are under the regulation of RbsR (SACTE_5759). The putative ligands of BxlR and RbsR are xylobiose and xylose, respectively. The red arrows indicate genes involved in xylan degradation. The green arrows indicate genes encoding transporters. The blue arrows identify transcriptional regulators, and the gray arrow indicates a protein unrelated to xylan degradation.

We mapped transcriptomic, proteomic, and biochemical analysis results to metabolic pathway models for SirexAA-E (see KEGG T01601, ssx). In combination with the transcriptomic data from our earlier study on xylan utilization (2), the present study gives a fuller picture of cellular responses to xylose, xylobiose, and xylan in the metabolic pathway from xylan to phosphoenolpyruvate (Fig. 6 and 7) and the subsequent TCA cycle (Fig. S10). The incorporation of both extra- and intracellular proteomic data sets provides further evidence for the metabolic specializations required for growth on pentose sugars derived from xylan.

Xylobiose is metabolized through the pentose phosphate pathway and glycolysis (Fig. 6). Other than xylose, longer xylooligosaccharides are hydrolyzed into xylose by two xylosidases, SACTE_0531, and SACTE_5159 (GH3), and isomerized by xylose isomerase (SACTE_5230), which was highly abundant in our proteome data sets. In the pentose phosphate pathway, the formation of glyceraldehyde 3-phosphate (G3P) from xylulose-5P requires two enzymes, ATP-dependent xylulokinase (COG: G; SACTE_5827 and SACTE_1916) and erythrose-4P-requiring transketolase (COG: G; among several, SACTE_0274, SACTE_0563, and SACTE_1355) (32, 33). Although the SACTE_5827 transcript was low in abundance (2), the enzyme was readily detected in all intracellular proteomes. Moreover, two transketolases (SACTE_0274 and SACTE_0563) were enriched in the mRNA transcript data set (2), while SACTE_1355 exhibited low, but detectable protein abundance in the xylobiose/glucose and xylan/glucose, and SACTE_0563 was weakly detected in the xylose/glucose proteome (Fig. 6). Presently, it appears these three detected transketolases may be subject to different regulatory control schemes but are otherwise functionally redundant. The transketolase products glyceraldehyde-3P (G3P) and fructose-6P (F6P) are substrates for glycolysis.

The enzymes for the conversion of G3P to phosphoenolpyruvate (PEP) found in the SirexAA-E proteome include three isoforms of G3P dehydrogenase (GAPDH, COG: G; SACTE_0169, SACTE_1369, and SACTE_5983), and one each of phosphoglycerate kinase (PGK, COG: G; SACTE_1368), phosphoglycerate mutase (PGM, COG: G; SACTE_3666), and enolase (COG: G; SACTE_2556) (Fig. 6). The corresponding transcripts and proteins were detected under all growth conditions, albeit weakly. This weak detection is likely due to normalization against the robust growth on glucose via glycolysis, compared to the alternative pathways used for pentose metabolism.

ATP provides chemical energy for many cellular functions, including protein and nucleic acid synthesis, protein secretion, sugar and oligosaccharide import. Along with ribosomal biogenesis, the synthesis of proteins consumes the major fraction of intracellular ATP (34, 35). For growth on extracellular polymers like xylan, the abundant synthesis and secretion of xylanases represent a substantial ATP demand (Fig. 4). Additionally, ATP hydrolysis is required for the import of mono- and oligosaccharide products into the cell for further metabolism (26). These demands likely account for the observed upregulation of numerous oxidative phosphorylation-related proteins during growth on xylan.

### Potential transcriptional regulators involved in xylan catabolism

The results from pull-down proteomics identified transcriptional regulators binding to the upstream regions of two secreted xylanases, SACTE_0265 (GH10) and SACTE_0358 (GH11), and intracellular xylose isomerase (SACTE_5230), all of which were highly enriched in the presence of xylobiose- or xylan-grown cells (Tables S9 through S15). The five transcriptional regulators were identified by the pull-down proteomics (Table 3). Two LacI-family regulators, SACTE_0535 for *bxl* and SACTE_5759 for *rbs1* clusters, respectively, were enriched in the presence of xylose, xylobiose, or xylan, perhaps by derepressing themselves in the presence of effector ligands such as xylose and xylobiose, as reported earlier (4, 8, 16, 36). An AsnC-like transcriptional regulator, SACTE_2715, can function as either an activator or repressor in *Streptomyces* species (37). A homolog of SACTE_2715 is present in the *S. coelicolor* genome, yet the function of this protein has not been determined (38). The PadR regulator in *S. coelicolor* has been identified as a regulator of cell differentiation (37, 39)*,* with a homolog SACTE_3328 found in SirexAA-E. An IclR-family regulator, SACTE_5479, might also function as a repressor as its homolog in *S. coelicolor* was shown to repress allantoin biosynthesis (40–42). Notably, SACTE_0535 and SACTE_5759, determined by the quantitative proteomics analysis, are a part of gene clusters encoding annotated sugar transporters (Fig. 5). Thus, SACTE_0535 and SACTE_5759 are thought to be key regulators for xylan response in SirexAA-E by upregulating genes encoding for extracellular enzymes, transporters, enzymes in metabolic pathways, and others (Fig. 7).

### Transcriptional versus post-transcriptional regulations in SirexAA-E

We described xylan-specific protein secretion, expression of transporters, metabolic regulation, and transcriptional response of SirexAA-E. Among the metabolic responses, we carried out intracellular proteomics to assess the levels of protein abundance in cells grown on xylose, xylobiose, or xylan relative to glucose-grown cells, along with the differentially expressed mRNAs determined in xylan-grown cells versus glucose-grown cells (Fig. 6, S9, and S10). Protein products such as secreted enzymes (SACTE_0265, SACTE_0357, and SACTE_0358), transporter (SACTE_0532-0534), intracellular β-xylosidase (SACTE_0531), and xylose isomerase (SACTE_5230) were found to be highly abundant, and their corresponding transcripts were also enriched (Fig. 6). However, the discrepancy between the abundance of transcripts and proteins was also observed in our data sets. For example, succinate dehydrogenase-coding mRNAs were overall poorly transcribed, while the levels of their products were highly enriched (Fig. S10). Our findings are consistent with the previous notions in broad organisms and even single cells of model bacteria *E. coli* (43, 44). Moreover, the small noncoding RNAs mediating post-transcriptional regulation of PEPCK, which converts oxaloacetate to phosphoenolpyruvate, were recently reported in *S. coelicolor* (45). Altogether, the current study suggests that metabolic regulation in this organism occurs not only at the transcriptional level but also through significant post-transcriptional regulation, such as alterations in transcripts and protein stability, or translation efficiency.

## MATERIALS AND METHODS

### Chemicals and reagents

Carbon sources, including D-glucose, D-xylose (Wako Pure Chemical Industry, Ltd., Osaka, Japan), xylobiose (Megazyme, Bray, Ireland), and xylan (Tokyo Chemical Industry Co., Ltd., Tokyo, Japan), were used. The synthetic M63 medium contained 5.24 g/L KH_2_PO_4_, 10.72 g/L K_2_HPO_4_, 2.0 g/L (NH_4_)_2_SO_4_, 1.0 mM of MgSO_4_, 1 mg/L thiamin, and 0.5% wt/vol of the designated carbon source.

### Cultivation of SirexAA-E

Cells were precultured in 2 mL of M63 liquid medium, containing 0.5% wt/vol of D-(+)-glucose, D-(+)-xylose, xylobiose, or xylan as a sole carbon source and grown for 3 days at 30°C at 250 rpm. A volume of 200 µL each of the precultured cells was inoculated into 20 mL of M63 liquid medium containing the same carbon sources used in the preculture (0.5% wt/vol). The cells were grown for 3 days at 30°C at 250 rpm, and three biological replicas from each carbon source for the extracellular proteomics and one biological replica for the intracellular proteomics were prepared. Cell growth on each culture condition was monitored by real-time PCR (Light Cycler 96, Roche, Mannheim, Germany) using extracted genomic DNA at different time points (24, 48, 72, and 120 hours) in triplicate (46). The Cq values were converted to the dry cell weight (mg per liter culture) by using a standard curve, as described previously (47). Briefly, SirexAA-E was grown on the M63 in the presence of 0.5% glucose for 3 days at 30 ˚C in a 4 mL culture scale, and 1 mL of the culture was used to extract genomic DNA for the Cqs, and the rest was used to weigh dried cells of SirexAA-E to correlate the Cq value and DCW.

### Preparation of extracellular proteins for proteomic analysis

The culture supernatants were centrifuged for 15 min at 4,000×*g* at 4°C to remove cells. The culture supernatant was filtered by using a 0.45 µm glass fiber filter (AS ONE Co., Osaka, Japan). Proteins were concentrated using centrifugal ultrafiltration (VIVASPIN 20; Sartorius AG, Göttingen, Germany) against 20  mM sodium phosphate (pH 7.4). The final protein concentration was estimated by a BCA protein assay kit (BioRad, Hercules, CA).

Sample preparation for proteomics analysis was performed as described elsewhere (8). Briefly, around ~ 20 µg of extracellular protein sample was precipitated by TCA and dissolved in 10 µL 8M urea. Seventy microliters of 50 mM ammonium bicarbonate was added, and reduction and alkylation reactions were performed followed by protease digestion using a 1:40 trypsin/protein ratio (proteomics grade; Roche). Samples were purified by C18 ZipTip pipette tips (Merck Millipore, Burlington, MA) and eluted in 0.1% formic acid in water. The extracellular proteomics was performed in three biological replicates.

### Cell lysate preparation and intracellular protein quantification

Cells grown on different carbon sources, including D-glucose, D-xylose, xylobiose, and xylan, were harvested by centrifugation for 15 min at 4,000×*g* at 4°C. The pellets were washed twice with 20 mM phosphate buffer, pH 7.4, and suspended in 500 µL of 20 mM phosphate buffer, pH 7.4, followed by sonication for 10 min on ice using Branson Sonifier 250 (Emerson, St. Louis, MO) at a 30% duty cycle and an output setting of 2. 1% wt/vol SDS was added, and the cell lysates were centrifuged for 30 min at 12,000×*g* at 4°C. The supernatants were collected into new tubes, and the total protein concentration was estimated by running a 10% stain-free SDS-PAGE (BioRad), followed by visualization using a Gel Dock system (BioRad).

### Preparation of intracellular proteome samples and TMT labeling

A 100 µg equivalent of the intracellular protein fraction from each culture, including the cells grown on D-glucose, D-xylose, xylobiose, and xylan, was suspended in 100 µL of 100 mM TEAB solution (Thermo-Fisher Scientific, Waltham, MA) and then reduced by adding 200 mM TCEP (Thermo-Fisher Scientific) for 1 h at 55°C, alkylated by adding 50 mM iodoacetamide for 30 min at room temperature in the dark, followed by precipitation by ice-cold acetone. The protein pellet was obtained by centrifugation for 10 min at 8,000×*g* at 4°C and air-dried, and then the pellet was resuspended in 50 mM TEAB solution. All samples were digested overnight using a 1:40 trypsin/protein ratio (proteomic grade; Roche) at 37°C, and the peptide concentration was measured using the Pierce Quantitative Colorimetric Peptide Assay kit (Thermo-Fisher Scientific).

TMT-duplex Isobaric Label Reagent (Thermo-Fisher Scientific) was pre-aliquoted at the concentration of ~ 20 µg/µL in anhydrous acetonitrile. Approximately 100 µg of peptide from the culture containing D-glucose was labeled using the lighter TMT reagent (TMT^2^-126), and 33.3 µg of the peptide from the cultures containing D-xylose, xylobiose, or xylan was labeled with the heavier TMT reagent (TMT^2^-127) for 1 h at room temperature. Then, 5% hydroxylamine (Thermo-Fisher Scientific) quenched the reaction, and 33.3 µg of the peptide from the culture containing D-glucose was combined with 33.3 µg each of TMT^2^-127 peptide from D-xylose, xylobiose, or xylan grown cells. Finally, samples were purified by using C18 ZipTip pipette tips (Merck Millipore) and eluted in 0.1% formic acid in water. Intracellular quantitative proteomic analysis was performed in two biological replicates and validated by correlation analysis.

### Proteomic analyses

Mass spectra were obtained using an Easy nLC1000 liquid chromatography system with Q-Exactive Plus Orbitrap mass spectrometer (Thermo Fisher Scientific) and Xcalibur software v. 3.1 (Thermo Fisher Scientific) for both extracellular and intracellular samples. The peptides were separated on a C18 capillary tip column (NTCC-360/75-3-125; Nikkyo Techno, Japan) by a linear gradient from 0% to 40% with two solutions—0.1% formic acid in water (solution A) and 0.1% formic acid in acetonitrile (solution B) for 120 min at a flow rate of 300 nL/min for extracellular samples, and linear gradient from 0 to 25% for 200 min and 25 to 60% for 40 min with solution A and solution B at the same flow rate for intracellular samples. Full-scan mass spectra were acquired with a scan range of 300.0 to 2,000.0 *m/z*, and 375.0 to 1,400.0 *m/z* at a resolution of 70,000 for extracellular and intracellular samples, respectively, with a maximum injection time of 50 ms and Auto Gain Control (AGC) 3e6. Proteomic identification and quantification were performed from the acquired MS/MS spectra using Proteome Discoverer v. 2.1 (Thermo Fisher Scientific) with all coding DNA sequences (CDSs) of SirexAA-E reported in the previous study (2). The precursor mass tolerance was set to 10 ppm, and fragment mass tolerance was set to 0.6 Da for the extracellular proteomic analysis. For the intracellular proteomic analysis, the precursor mass tolerance and fragment mass tolerance were set to 10 ppm and 0.8 Da, respectively. The peptide charge was set at + 2, +3, and + 4 for both analyses. The accuracy and sensitivity of peptide identification were optimized using the automatic decoy and percolator functions built into the Proteome Discoverer software.

### Intracellular ATP and ADP concentrations

The cells of SirexAA-E were cultivated in 30 mL M63 medium containing 0.5% D-glucose, D-xylose, or xylan for 3 days at 30°C, and cells were harvested by centrifugation at 4,000×*g* for 10 min at 4°C. A portion of the culture was taken to determine dry cell weight, and the remainder was used to determine the intracellular concentration of ATP and ADP by means of the ADP/ATP ratio assay kit (Dojindo, Kumamoto, Japan). Cells equivalent to ~ 0.25 mg were washed once with M63 medium and used immediately as the substrate in luciferase assays to determine the concentration of ATP and ADP using a Lumat LC9807 luminometer (Berthold, Nashua, NH). Luminescence intensity in each reaction was normalized by using the dry cell weight. Three biological replicas were performed for each reaction.

### Bioinformatics

Predicted protein functions were classified using 26 COGs, and violin plots and Spearman correlation analyses were created by R package ver. 4.2.2. A metabolic map of SirexAA-E was drawn by using the KEGG pathways, #ssx00010, #ssx00030, #ssx00040, #ssx00230, #ssx00340, and #ssx01100 (https://www.genome.jp/kegg/pathway.html). The abundance of proteins assigned to these metabolic pathways and gene clusters was obtained from a pair-wise TMT proteomics for xylose/glucose, xylobiose/glucose, and xylan/glucose and standardized relative to five DNA polymerases, including SACTE_1423, SACTE_1493, SACTE_1975, SACTE_2990, SACTE_3307, and SACTE_3492 (Table S16). RNA profiles revisited and used to construct metabolic pathways in this study were originally reported in the previous study (2).

### Pull-down proteomics

Cells were grown in the M63 supplemented with 0.5% glucose or xylan for 3 days at 30 ˚C. Intracellular proteomes were extracted as described in the quantitative intracellular proteomics method. From 350 to 550 bp of the 5'-biotinylated DNA fragments corresponding to three promoter regions for SACTE_0265, SACTE_0358, and SACTE_5230 were prepared by using 5’-biotinylated forward primers (Eurofins Genomics, Tokyo, Japan) and non-labeled reverse primer pairs (Fasmac, Kanagawa, Japan), and the SirexAA-E genome was used as a template, followed by gel purification (Kanto Kagaku, Tokyo, Japan) (Table S17). Biotin-based pull-down proteomics analysis was conducted as described in the previous study with slight modifications (48). Fifty micrograms of the intracellular proteins was mixed with 250 ng of biotinylated DNA fragments in 500 µL of binding buffer containing 200 mM NaCl, 0.1% of Tween20, 1 mM EDTA, and 20 mM Tris-HCl (pH 7.4) along with 50 µg of BSA and 2.5 mg of ~ 500 bp sheared SirexAA-E genomic DNA prepared by sonication to prevent nonspecific binding. The mixtures were incubated for 60 min at 15°C with moderate rotation, followed by adding 50 µg of streptavidin magnetic beads (Magnosphere, JSR Life Sciences Co, Ibaraki, Japan), and incubated for 15 min at 15°C. Then, the streptavidin magnetic beads were washed 10 times using 200 µL binding buffer. The pull-downed proteins were suspended in 10 µL of 8M urea. The following treatment for proteomics and measurement settings was the same as the extracellular proteomics analysis. Resulted proteins were sorted with protein abundance estimated by Sequest HT using Proteome Discoverer v. 2.1 (Thermo Fisher Scientific) with high FDR from three biological replicas, and predicted transcriptional regulators were selected. Among the top 15 transcriptional regulators that were highly enriched in the xylan data sets and absent from the glucose data sets for all, three target promoter regions were selected as xylan-responsive regulators. The original list of transcriptional regulators is available (Tables S9 through S15). The pull-down proteomic analysis was performed in three biological replicates.

## Data Availability

All proteomic data sets obtained in this study can be accessed via the PRIDE proteomics identification database (https://www.ebi.ac.uk/pride/) under accession number PXD067972 for extracellular and intracellular proteomics analysis and PXD061680 for pull-down proteomics analysis.

## SUPPLEMENTAL MATERIAL

Supplemental figures spectrum.02251-25-s0001.docx Figures S1 to S11.

Tables S1 to S4 spectrum.02251-25-s0002.xlsx Extracellular proteomics.

Tables S5 to S8 spectrum.02251-25-s0003.xlsx Quantitative intracellular proteomics.

Tables S9 to S15 spectrum.02251-25-s0004.xlsx Pull-down proteomics.

Table S16 spectrum.02251-25-s0005.xlsx Polymerases used for correction in heat map.

Table S17 spectrum.02251-25-s0006.docx Primers used for pull-down proteomics.

ASM does not own the copyrights to Supplemental Material that may be linked to, or accessed through, an article. The authors have granted ASM a non-exclusive, world-wide license to publish the Supplemental Material files. Please contact the corresponding author directly for reuse.
