# Supplementary material for: Multi-proteomics reveals integrated metabolic and regulatory networks for xylan catabolism in Streptomyces sp. SirexAA-E
Source: Microbiol Spectr. 2025 Nov 20;14(1):e02251-25. doi: 10.1128/spectrum.02251-25 (PMC12772271; doi:10.1128/spectrum.02251-25)
Supplement: Supplemental figures — Figures S1 to S11. [file spectrum.02251-25-s0001.docx]

Supplementary figures

**Multi-proteomics reveals integrated metabolic and regulatory networks for xylan catabolism in *Streptomyces* sp. SirexAA-E**

Tatsuya Nagano ^1,2^, Keisuke Ohashi ^1,2^, Petra Banko ^2^, Vijay Kumar ^2,3^, Chiaki Hori ^4^, Brian G. Fox ^5,6^, Taichi E. Takasuka^1,2,6^#

^1^Graduate School of Global Food Resources, Hokkaido University, Sapporo, Japan

^2^Research Faculty of Agriculture, Hokkaido University, Sapporo, Japan

^3^Department of Microbiology, Guru Nanak Dev University, Amritsar-143005, Punjab, India

^4^Environmental Molecular Biology, Section of Environmental Biology, Faculty of Environmental Earth Science, Hokkaido University, Sapporo, Japan

^5^Department of Biochemistry, University of Wisconsin-Madison, Madison, Wisconsin, USA

^6^Global Station for Food, Land and Water Resources, Hokkaido University, Sapporo, Japan

#Address correspondence to Taichi E. Takasuka, takasuka@agr.hokudai.ac.jp


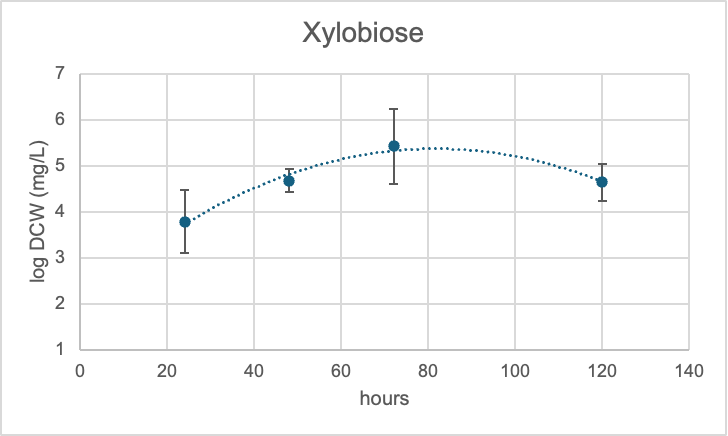

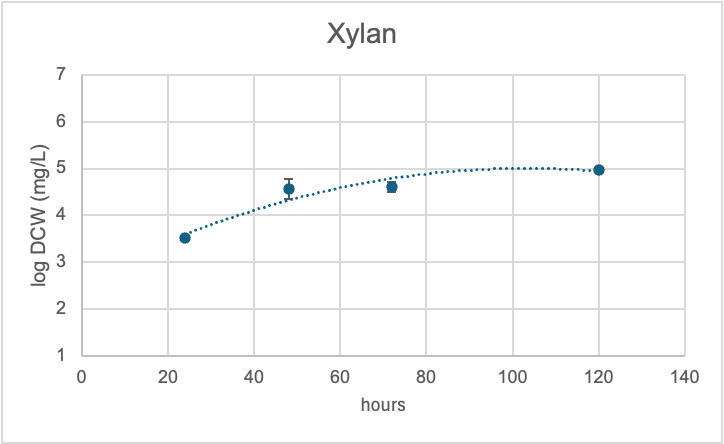

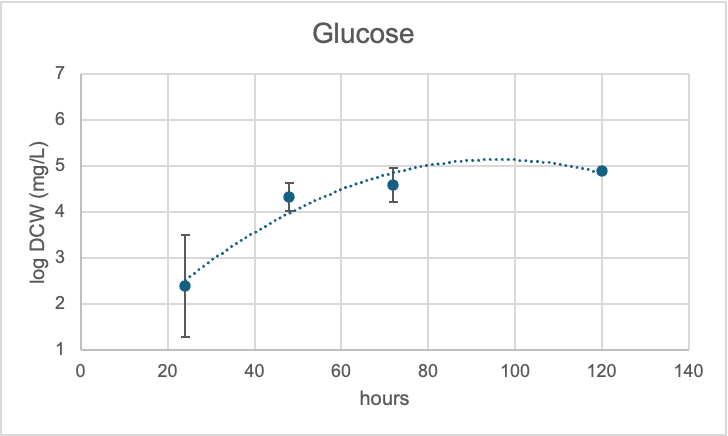

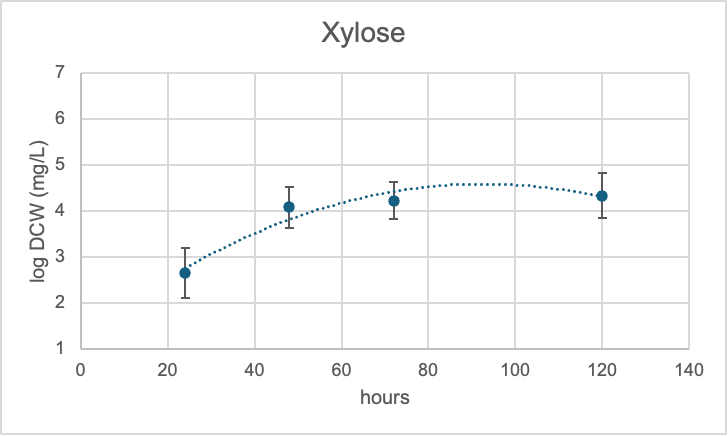


**Figure S1**. Growth of SirexAA-E cells on different carbon sources, including glucose, xylose, xylobiose and xylan. Cells were collected at 24, 48, 72 and 120 h. After each time point, cells were harvested and the genome DNA was extracted for qRT-PCR. The obtained Cq value was converted to DCW (mg/mL) by the standard curve correlating the DCW and Cq. The experiment was performed in triplicate, and the standard deviation was calculated from 3 biological replicates.


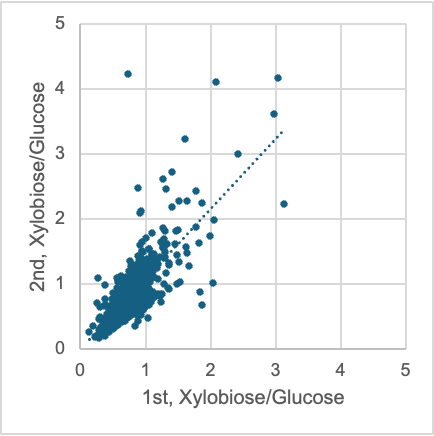

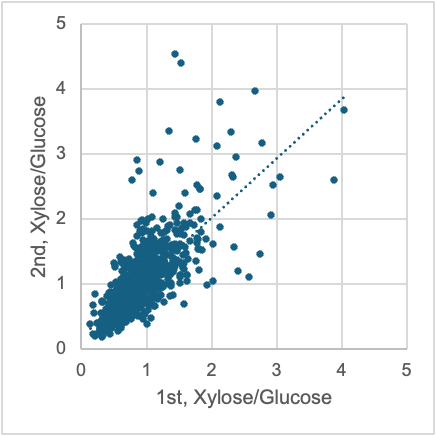

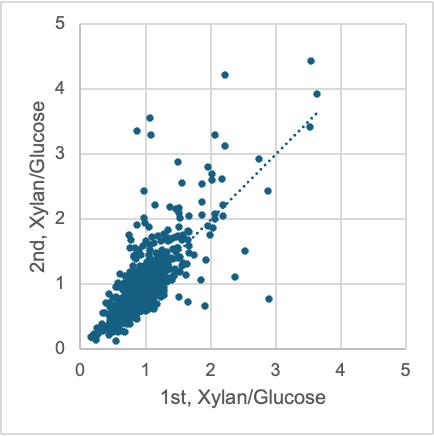


R=0.72, p<2.6e-235

Sample Size: 1463

R=0.78, p<4.3e-277

Sample Size: 1344

R=0.74, p<4.5e-215

Sample Size: 1257

**Figure S2.** Spearman’s correlation plot of intracellular proteins between duplicates of the quantitative intracellular proteomics for xylose/glucose, xylobiose/glucose, and xylan/glucose. Each axis shows the protein abundance relative to glucose-grown cells determined by quantitative proteomics. The dotted line dictates the fitted linear regression. *R* represents Spearman’s correlation coefficient, and the *P*-value shows the significance of the correlation in the upper left corner. Overall, two independent biological replicas show statistically significantly high correlations for each comparison, which validate our intracellular proteomics results.

**Figure S3.** Venn diagram showing the number of proteins determined by quantitative intracellular proteomics in xylose/glucose, xylobiose/glucose, and xylan/glucose datasets.

**
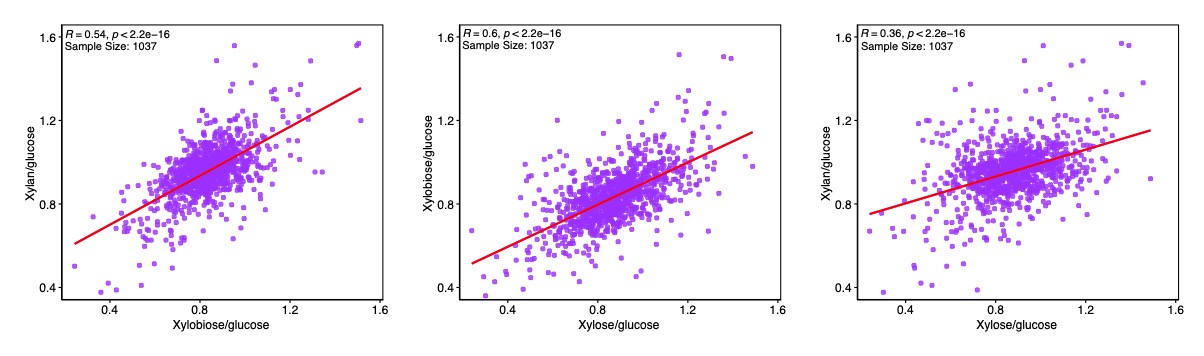
**

**Figure S4.** Spearman’s correlation plot of 1037 intracellular proteins found in all three samples (Fig. S3) between Xylan/glucose versus Xylobiose/glucose, Xylobiose/glucose versus Xylose/glucose, and Xylan/glucose versus Xylose/glucose. Each axis shows protein abundance relative to glucose-grown cells as determined by quantitative proteomics. The red line indicates the fitted linear regression. *R* denotes Spearman’s correlation coefficient, and the *P*-value indicates the significance of the correlation in the upper left corner, respectively.

**
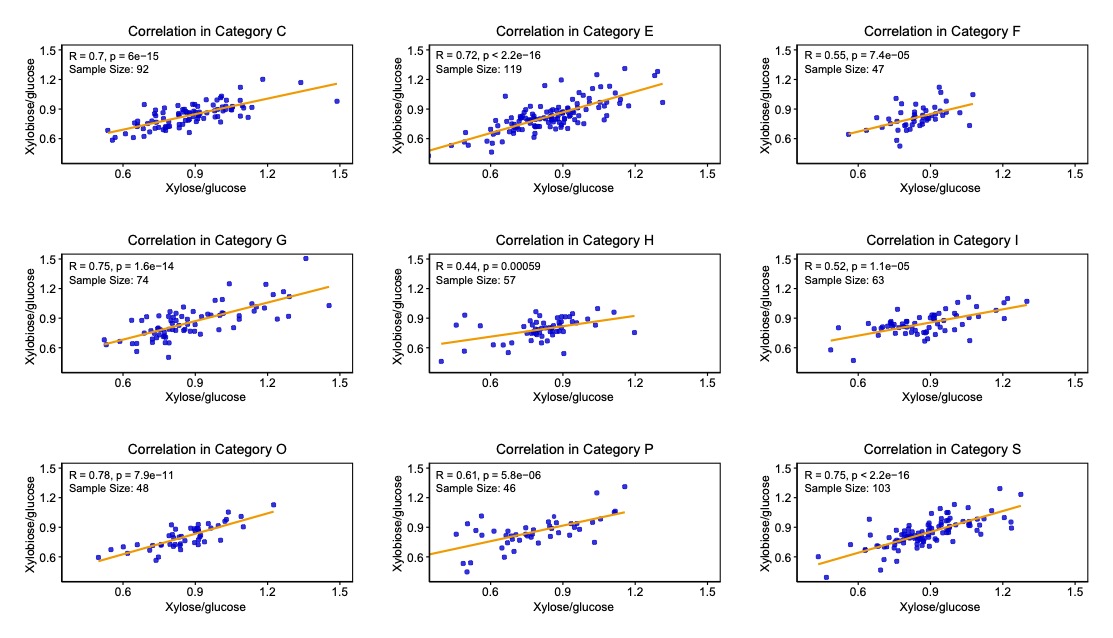
**

**Figure S5.** Spearman’s correlation plot of intracellular proteins in nine COG categories (C, E, F, G, H, I, O, P, S). Abundances of Xylose/glucose and Xylobiose/glucose are used for the comparison to confirm positive correlations. The orange line illustrates the fitted linear regression. *R* represents Spearman’s correlation coefficient, and the *P*-value shows the significance of the correlation in the upper left corner, respectively.

**
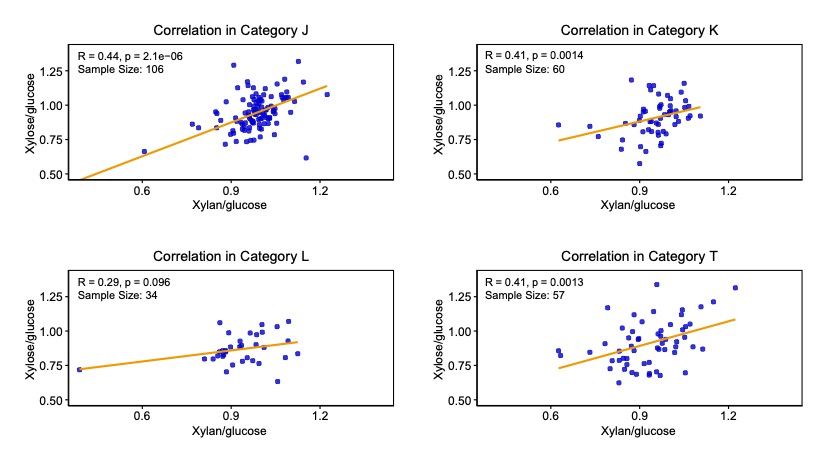
**

**Figure S6.** Spearman’s correlation plot of intracellular proteins in four COG categories (J, K, L, T). Abundances of Xylose/glucose and Xylan/glucose are used for the comparison to confirm positive correlations. The orange line illustrates the fitted linear regression. *R* represents Spearman’s correlation coefficient, and the *P*-value shows the significance of the correlation in the upper left corner, respectively.

**
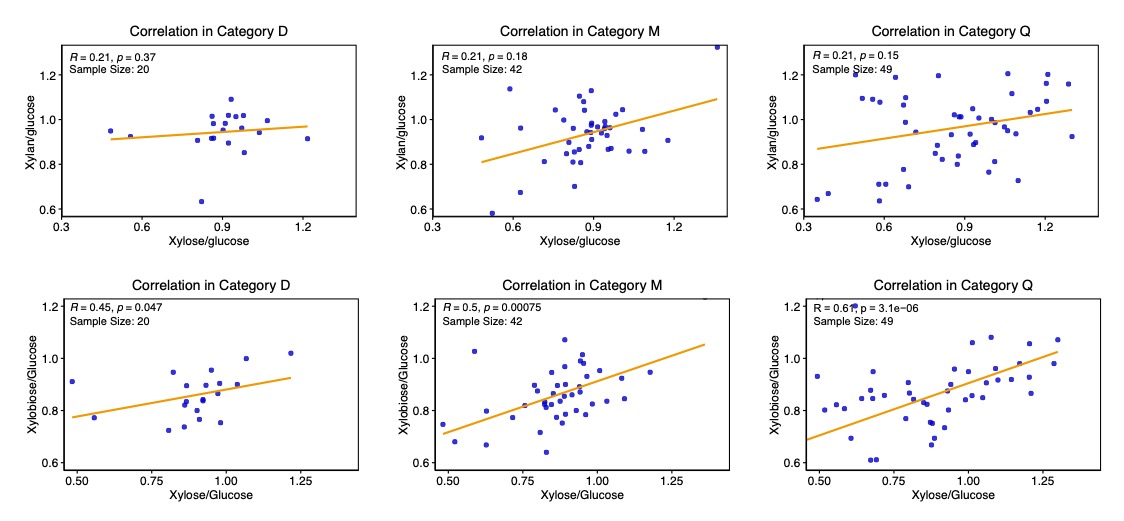
**

**Figure S7.** Spearman’s correlation plot of intracellular proteins in three COG categories (D, M, Q). Abundance combinations of Xylan/glucose versus Xylose/glucose and xylobiose/glucose versus xylose/glucose are used for this comparison to confirm positive correlations. The orange line illustrates the fitted linear regression. *R* represents Spearman’s correlation coefficient, and the p-value shows the significance of the correlation in the upper left corner, respectively.


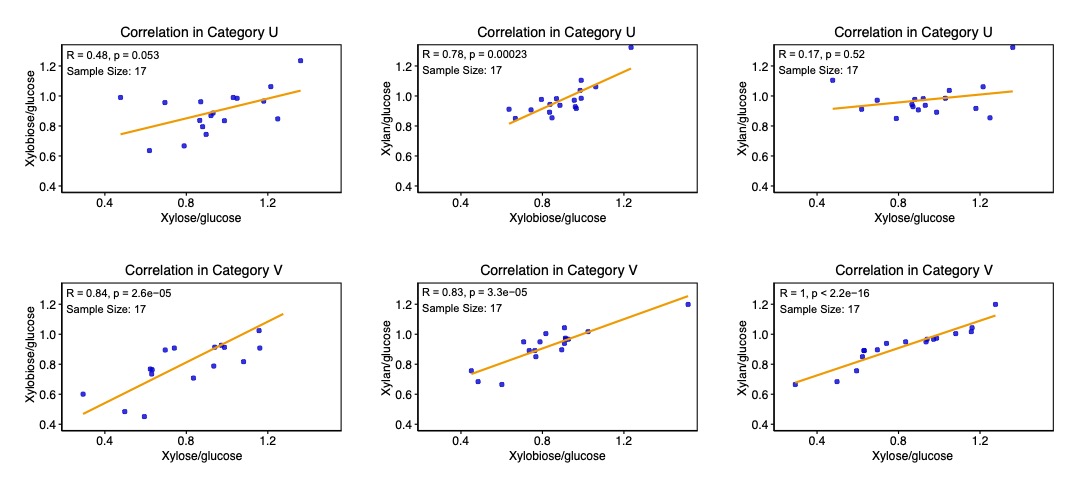


**Figure S8.** Spearman’s correlation plot of intracellular proteins in two COG categories (U, V). Abundance combinations of Xylobiose/glucose vs. Xylose/glucose, Xylan/glucose vs. Xylobiose/glucose and Xylan/glucose vs. Xylose/glucose are used for this comparison to confirm positive correlations. The orange line illustrates the fitted linear regression. *R* represents Spearman’s correlation coefficient, and the p-value shows the significance of the correlation in the upper left corner, respectively.


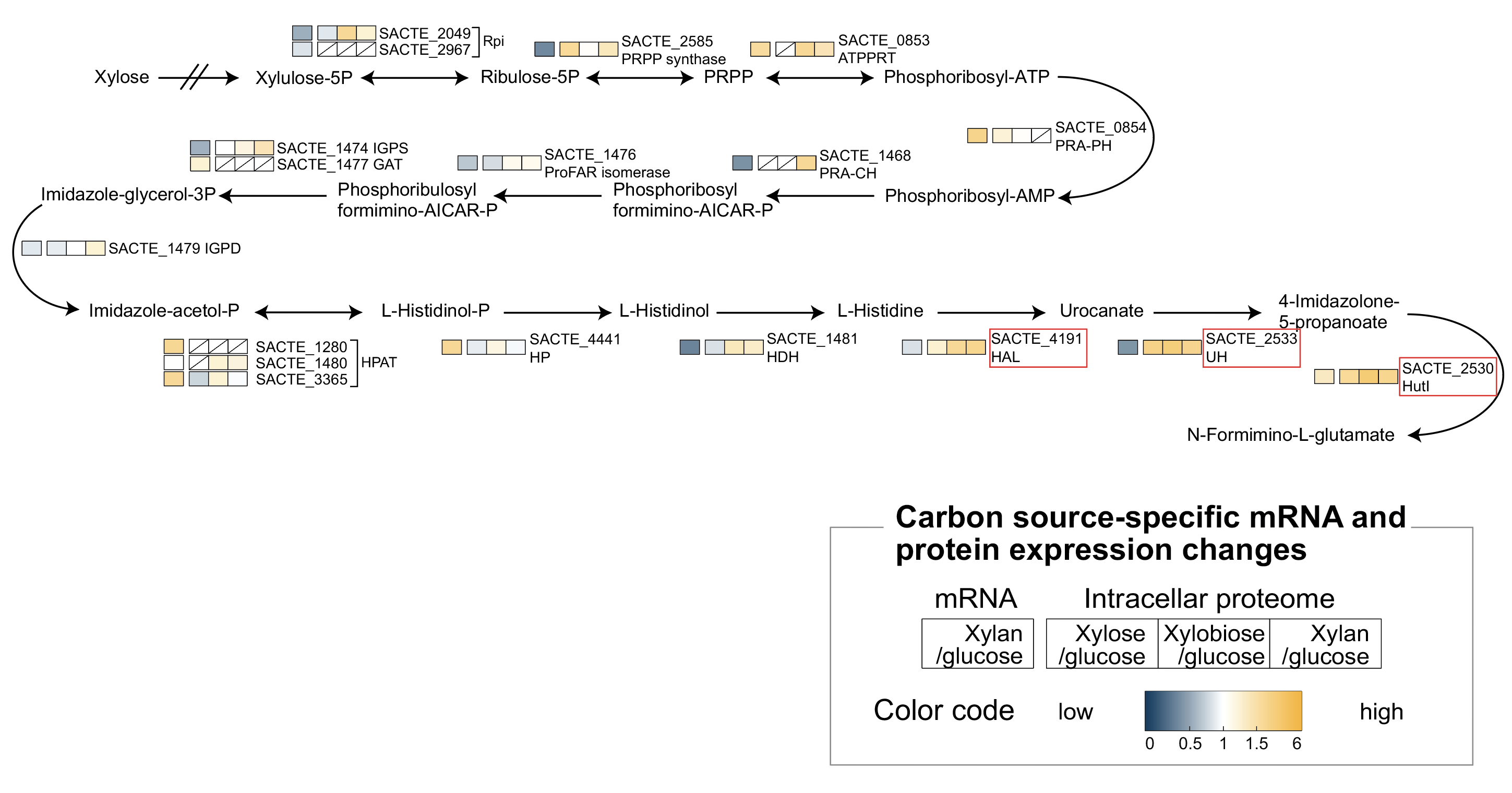


**Figure S9.** Additional KEGG pathways, including the synthesis and degradation of histidine. The KEGG pathway (Pathways #ssx00030 and #ssx00340) was built and enzymes that catalyze indicated reactions were overlayed with the corresponding xylan/glucose transcriptome dataset, xylose/glucose, xylobiose/glucose and xylan/glucose intracellular proteome datasets. The relative abundance of mRNA and proteins in each dataset were calculated and color-coded. The square with diagonal line indicates proteins that were not quantified. Enzymes mentioned in the main text are highlighted with red squares. Abbreviations: Rpi, ribose 5-phosphate isomerase; PRPP, Phosphoribosyl pyrophosphate; ATPPRT, ATP phosphoribosyltransferase; PRA-PH, phosphoribosyl-ATP pyrophosphatase; PRA-CH, phosphoribosyl-AMP cyclohydrolase; ProFAR, 1-(5-phosphoribosyl)-5-[(5-phosphoribosylamino)methylideneamino] imidazole-4-carboxamide; IGPS, imidazole glycerol phosphate synthase; GAT, glutamine amidotransferase; IGPD, imidazoleglycerol-phosphate dehydratase; HPAT, histidinol-phosphate aminotransferase; HP, histidinol-phosphatase; HDH, histidinol dehydrogenase; HAL, histidine ammonia-lyase; UH, urocate hydratase; HutI, imidazolonepropionase.

**
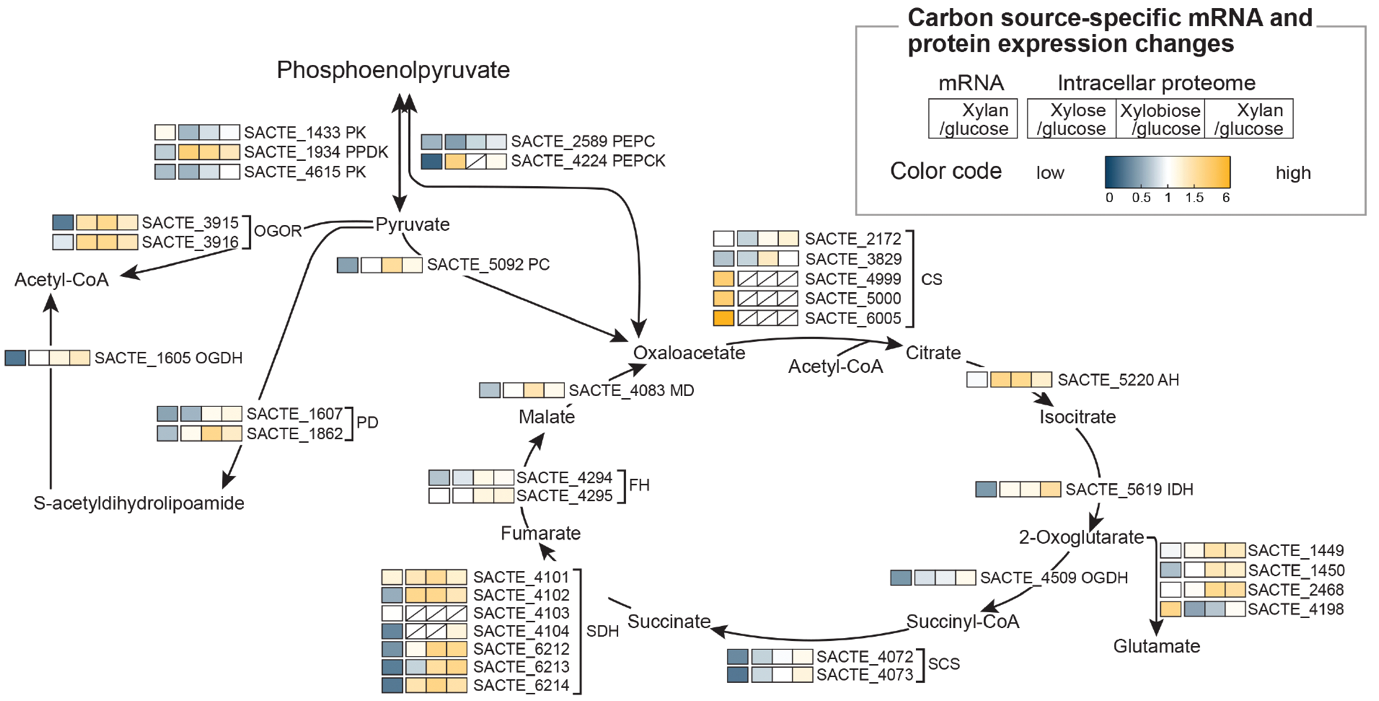
**

**Fig. S10.** Xylose, xylobiose, and xylan specific responses in representative metabolic pathways from the final steps of glycolysis to the TCA cycle and acetyl CoA synthesis. The levels of corresponding transcripts (Xylan/glucose) and quantified enzymes from present intracellular proteome datasets are shown with the color code. Squares with a diagonal line indicats proteins that were not quantified or detected by proteomics. Abbreviations: PK, pyruvate kinase; PPDK, pyruvate phosphate dikinase; PEPC, phosphoenolpyruvate carboxylase; PEPCK, phosphoenolpyruvate carboxykinase; OGOR, 2-oxoglutarate ferredoxin oxidoreductase; PD, pyruvate dehydrogenase; OGDH, 2-oxoglutarate dehydrogenase; PC, pyruvate carboxylase; CS, citrate synthase; AH, aconitase; IDH, isocitrate dehydrogenase; OGDH, 2-oxoglutarate dehydrogenase; ALT, alanine-synthesizing transaminase; AST, L-aspartate aminotransferase; SCS, succinyl-CoA synthetase; SDH, succinate dehydrogenase; FH, fumarase; MD, malate dehydrogenase.

**
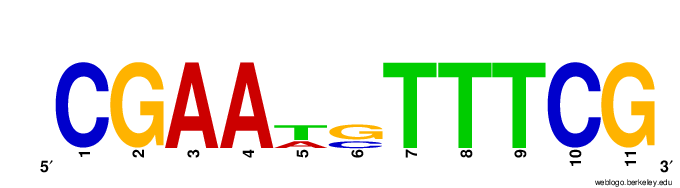
**

**Fig. S11.** Multiple sequence alignment of putative SACTE_0535 binding sites created by Weblogo (https://weblogo.berkeley.edu/logo.cgi). The sequence motif was determined in the P0265, P0358, and P0535 regions.
