# Supplementary material for: Multi-proteomics reveals integrated metabolic and regulatory networks for xylan catabolism in Streptomyces sp. SirexAA-E
Source: Microbiol Spectr. 2025 Nov 20;14(1):e02251-25. doi: 10.1128/spectrum.02251-25 (PMC12772271; doi:10.1128/spectrum.02251-25)
Supplement: Table S17 — Primers used for pull-down proteomics. [file spectrum.02251-25-s0006.docx]

Table S17. Primers used to amplify the promoter regions for pull-down proteomics

|  |  |
| --- | --- |
| Name | Sequence |
| Prom_SACTE0265_fw | 5’-CGCCGCACGTCACGGTCGTTG-3’ |
| Prom_SACTE0265_rv | 5’-CGGTCTGTGGACCGTGGGCTGG-3’ |
| Prom_SACTE0357_fw | 5’-CAGGTCGTCGACATGTGGCAGGC-3’ |
| Prom_SACTE0357_rv | 5’-AGTGCTCGCCGGGGGTGACGG-3’ |
| Prom_SACTE5230_fw | 5’-CCGCGTCGTCAAGATCGTGCGG-3’ |
| Prom_SACTE5230_rv | 5’-TTGCCGTCGGAGGTGGCGATGCG-3’ |
